# Cell wall biogenesis of *Arabidopsis thaliana *elongating cells: transcriptomics complements proteomics

**DOI:** 10.1186/1471-2164-10-505

**Published:** 2009-10-31

**Authors:** Elisabeth Jamet, David Roujol, Hélène San-Clemente, Muhammad Irshad, Ludivine Soubigou-Taconnat, Jean-Pierre Renou, Rafael Pont-Lezica

**Affiliations:** 1 Surfaces cellulaires et Signalisation chez les Végétaux, UMR 5546 CNRS - UPS -Université de Toulouse, Pôle de Biotechnologie Végétale, 24 chemin de Borde-Rouge, BP 42617 Auzeville, 31326 Castanet-Tolosan, France; 2 UMR INRA 1165 - CNRS 8114 - UEVE, 2 rue Gaston Crémieux, CP 5708, 91057 Evry, France; 3 Department of Botany, Govt. Post-Graduate College, 23200 Mardan, Pakistan

## Abstract

**Background:**

Plant growth is a complex process involving cell division and elongation. *Arabidopsis thaliana *hypocotyls undergo a 100-fold length increase mainly by cell elongation. Cell enlargement implicates significant changes in the composition and structure of the cell wall. In order to understand cell wall biogenesis during cell elongation, mRNA profiling was made on half- (active elongation) and fully-grown (after growth arrest) etiolated hypocotyls.

**Results:**

Transcriptomic analysis was focused on two sets of genes. The first set of 856 genes named cell wall genes (CWGs) included genes known to be involved in cell wall biogenesis. A significant proportion of them has detectable levels of transcripts (55.5%), suggesting that these processes are important throughout hypocotyl elongation and after growth arrest. Genes encoding proteins involved in substrate generation or in synthesis of polysaccharides, and extracellular proteins were found to have high transcript levels. A second set of 2927 genes labeled secretory pathway genes (SPGs) was studied to search for new genes encoding secreted proteins possibly involved in wall expansion. Based on transcript level, 433 genes were selected. Genes not known to be involved in cell elongation were found to have high levels of transcripts. Encoded proteins were proteases, protease inhibitors, proteins with interacting domains, and proteins involved in lipid metabolism. In addition, 125 of them encoded proteins with yet unknown function. Finally, comparison with results of a cell wall proteomic study on the same material revealed that 48 out of the 137 identified proteins were products of the genes having high or moderate level of transcripts. About 15% of the genes encoding proteins identified by proteomics showed levels of transcripts below background.

**Conclusion:**

Members of known multigenic families involved in cell wall biogenesis, and new genes that might participate in cell elongation were identified. Significant differences were shown in the expression of such genes in half- and fully-grown hypocotyls. No clear correlation was found between the abundance of transcripts (transcriptomic data) and the presence of the proteins (proteomic data) demonstrating (i) the importance of post-transcriptional events for the regulation of genes during cell elongation and (ii) that transcriptomic and proteomic data are complementary.

## Background

Plant growth occurs mainly by division and expansion of cells. A meristematic cell might enlarge as much as 50000-fold its initial volume. In this process, membrane surface area and amount of cell wall material increase. The primary cell wall plays an essential role since it should allow turgor-driven increase in cell volume by permitting the incorporation of new cell wall material and rearrangement of the existing cell wall. Several plant organs including coleoptiles (*poaceae*), internodes (legumes), and hypocotyls (mung bean, sunflower, and *Arabidopsis thaliana*) were used to study cell elongation [1]. Environmental signals such as light, temperature, and hormones, regulate hypocotyl growth [2-5]. *A. thaliana *seedlings grown in continuous darkness are a material of choice to analyze the cell elongation process. Indeed, cells of hypocotyls undergo a 100-fold length increase compared to embryo cells [6]. Growth occurs mostly by cell expansion, with little cell division [4,6-8]. Changes in wall thickness during elongation of *A. thaliana *hypocotyls were investigated using cryo-field-emission scanning electron microscopy [1]. At the germination stage, cell wall thickening occurs and involves high rates of biosynthesis and deposition of cell wall components. During the elongation stage, cell walls undergo remarkable thinning, requiring extensive polymer disassembly and rearrangement.

Many genes are assumed to be involved in cell wall synthesis and rearrangement to support growth of plant cell walls [9]. They encode cellulose synthases (CESAs), cellulose synthases-like (CSLs), endo-glucanases, xyloglucan endotransglucosylase/hydrolases (XTHs) and expansins. They belong to multigenic families, but the members of each family involved in elongation of hypocotyl cells were not precisely identified. It is also likely that other genes are important for cell elongation.

In this paper, the transcriptomes of *A. thaliana *etiolated hypocotyls were compared at two developmental stages, half-grown (yet actively elongating) and fully-grown (after growth arrest). The transcriptome analysis was focused on genes possibly involved in cell wall biogenesis and on genes encoding secreted proteins. Transcript profiling was carried out using CATMA (Complete *Arabidopsis *Transcriptome MicroArray) [10]: (i) to look at the level of transcripts of cell wall genes (CWGs) belonging to families known to be involved in cell wall biogenesis; (ii) to identify genes encoding secreted proteins (SPGs) having high or moderate level of transcripts; (iii) to reveal differential gene expression affecting CWGs and SPGs between half- and fully-grown etiolated hypocotyls; (iv) and to look at the correlation between transcript abundance and protein presence as revealed by a proteomic study performed on the same material [11].

## Results and Discussion

### Levels of transcripts of cell wall genes (CWGs) during hypocotyl elongation

**Figure 1:**
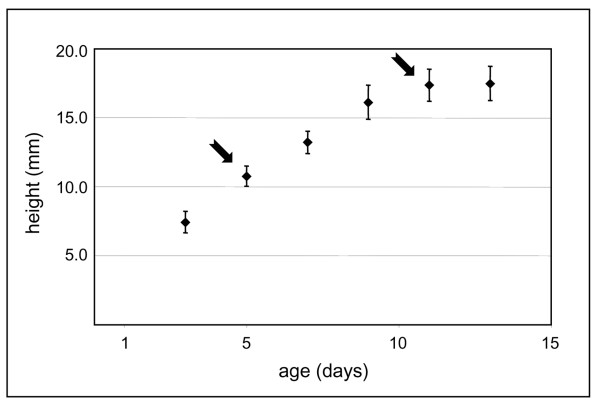
**Hypocotyl growth curve**. The height of dark-grown hypocotyls was measured every 2 days. Two stages were selected: 5- and 11-days (arrows), corresponding to half- (active elongation) and fully-grown (after growth arrest) etiolated hypocotyls respectively.

Etiolated hypocotyls were compared at two developmental stages. Five-day-old hypocotyls were approximately half the final size (Figure 1). Growth followed an acropetal gradient. After 5-days, the bottom cells were fully elongated, whereas the top cells were only starting elongation [8]. Eleven-day-old hypocotyls had reached their maximum size [6]. CATMA was used for mRNA profiling. Since one of the major modifications during cell elongation is the addition and rearrangement of cell wall components, a selection of genes possibly involved in cell wall biogenesis was done (Additional file 1). This selection was called "Cell Wall Genes" (CWGs). It was mainly based on the knowledge of gene families known to be involved in biogenesis of cell walls, *i.e*. synthesis and transport of cell wall components and their assembly or rearrangement in cell walls (see Methods). Representing 37 gene families, it includes genes encoding proteins involved in substrate generation (nucleotide-sugar inter-conversion pathway, monolignol biosynthesis), polysaccharide synthesis (mainly glycosyl transferases), vesicle trafficking, assembly/disassembly of the wall (glycoside hydrolases, expansins, carbohydrate esterases, carbohydrate lyases), structural proteins, oxido-reductases involved in cross-linking of wall components (mainly peroxidases and laccases). Few other gene families encoding cell wall proteins were also included such as arabinogalactan proteins (AGPs), fasciclin AGPs (FLAs), phytocyanins, multicopper oxidases, pectin methylesterase inhibitors (PMEIs), and subtilases. Only genes annotated by experts were retained (see Methods).

Altogether, 1026 genes were selected among which 856 were analyzed through CATMA, while the remaining genes were not analyzed for technical reasons. Some genes had no gene-specific tag (GST) on the microarray, others were not considered because of a poor signal of hybridization to the RNA probe, or of inaccurate duplicates. The level of transcripts was expressed as log_2 _of the mean signal intensity. Values of log_2 _below 6.83 were considered as background as defined in Methods. Three groups of genes were considered after taking into account the dynamic range of CATMA arrays [12]. Values between 6.83 and 9 corresponded to low level of transcripts (1- to 4-fold the background level), values between 9 and 10 to moderate level (4- to 8-fold the background level) and values higher than 10 to high level (more than 8-fold, and up to 128-fold the background level). Among the 856 genes analyzed, the level of transcripts at one or both stages was below background for 381 genes (44.6%), low for 326 (38.6%), moderate for 62 (7.2%) and high for 82 (9.6%).

Overall, among the analyzed genes, 49.4% of those related to synthesis or transport of cell wall components, and 52.2% of those involved in their modifications in cell walls have detectable level of transcripts. Among the 82 genes with high levels of transcripts at one or both stages (Additional file 2), 21 are involved in the synthesis or transport of cell wall components and 35 are involved in modifications of cell wall components. Two separate phases of growth were described in dark-grown *A. thaliana *hypocotyls: an early phase of active synthesis of cell wall polysaccharides up to 3-days after beginning of germination, and a late phase of cell expansion [8]. The former phase results in thicker cell walls which later on become thinner as hypocotyls elongate [1]. Our results suggest that both synthesis and rearrangement of cell wall components are required throughout hypocotyl elongation, and even after growth arrest.

Most of the CWGs were expected to be transcribed during cell elongation. Genes involved in rearrangement of cell wall components encode glycoside hydrolases (GHs) such as endoglucanases, XTHs, and beta-galactosidases; carbohydrate esterases (CEs) such as pectin methylesterases (PMEs); pectin acylesterases; polysaccharide lyases (PLs); expansins of the alpha- or beta-type; and peroxidases. However, 20 genes encoding PMEs and 5 genes encoding pectin acylesterases have detectable levels of transcripts among which 4 genes have high levels of transcripts in elongating hypocotyls (Additional files 1 and 2). This result seems contradictory with previous results showing that a low level of pectin esterification, associated to abundance of PMEs and acylesterases, restricted cell elongation in *A. thaliana *hypocotyls [13]. It should be noted that 18 genes encoding PMEIs also have detectable levels of transcripts. The interplay between PMEs and their inhibitors could regulate the activity of PMEs. Fifteen genes encoding proteins possibly involved in oxido-reduction reactions have high levels of transcripts (peroxidases, laccases, phytocyanins, and protein homologous to SKU5). Seven peroxidase genes fall in this category, among which *AT2G37130 *(AtPrx21) has one of the highest levels of transcripts. The multiple roles of peroxidases during growth and growth arrest were reviewed [14].

Other CWGs are also well-represented such as those encoding arabinogalactan proteins (AGPs), fasciclin AGPs (FLAs) and subtilases (Additional file 2). Even if AGPs and FLAs were shown to be associated with wood formation in poplar [15], their role in cell expansion is not very clear at present. Likewise, nothing is known about the role of proteases during cell elongation. Finally, the *COBRA *gene (*AT5G60920*) has a high level of transcripts. It has been shown to play an important role in microfibril deposition during rapid elongation and in the orientation of cellulose microfibrils [16].

This work gives clues for understanding the function and possible involvement in multiple processes of members of multigene families either during cell elongation or after its arrest. Indeed, general functions were proposed for most of these gene families, but only scarce information is available for specific members.

### Genes encoding secreted proteins with high or moderate level of transcripts in etiolated hypocotyls

Most of the gene families described above was already known to be involved in cell wall biogenesis. In order to identify new genes encoding secreted proteins that might be involved in cell expansion, a second selection was carried out. Based on results of proteomic studies, some proteins without a predicted signal peptide were assumed to be secreted. However, the sub-cellular localization of such proteins was never shown in another way [17]. For this study, only the genes encoding proteins with a predicted signal peptide were selected. The 2927 selected genes were ranked by level of transcripts, producing a profile similar to the one obtained with CWGs at 5-days; 1161 genes (39.7%) above background level, 1295 (44.2%) with a low transcript level, 238 (8.1%) with a moderate level, and 235 (8.0%) with a high level. Same results were obtained at 11-days. From this selection, only genes encoding proteins predicted to be located either outside the cell or in the plasma membrane were retained (see Methods). In Additional file 3, 433 genes named "Secretory Pathway Genes" (SPGs) with moderate or high levels of transcripts are listed and grouped in families according to their predicted functional domains (see Methods). Such protein families were already described in cell wall proteomic studies [17-19]: proteins acting on carbohydrates (69 genes); proteases (37 genes); proteins possibly involved in signaling (44 genes); structural proteins (15 genes); proteins possibly involved in oxido-reduction reactions (27 genes); proteins with interacting domains (33 genes); proteins related to lipid metabolism (40 genes); miscellaneous proteins (43 genes); proteins of unknown function (125 genes). Main differences between transcriptomic and proteomic data lie in the genes encoding proteins possibly involved in signaling since they comprise AGPs, FLAs, and plasma membrane proteins that are difficult to isolate, separate or identify through proteomics [18]. In the same way, the group of proteins of unknown function is very important because 48% of them are predicted to have trans-membrane domains. On the contrary, the group of structural proteins is probably under-represented because of the lack of appropriate GSTs for many of them. Indeed, their repetitive amino acid sequences make the design of specific probes difficult. One should note the abundance of proteases that can be assumed to be essential for protein turnover in tissues undergoing rapid elongation followed by elongation arrest within a short time. They may also be involved in signaling [20,21] or in protein maturation [22]. In addition, there are probably interactions between proteases and protease inhibitors to regulate the proteolytic activities in cell walls. Among the 125 proteins of yet unknown function, 25 have known structural domains. Others share domains with other proteins, such as domains of unknown function (DUF), or belong to the so-called uncharacterized protein families (UPF). Many are of particular interest, since they are only present in plants.

Among these 433 SPGs, only the 69 encoding proteins acting on carbohydrates, and the 12 encoding peroxidases or laccases were shown or assumed to contribute to assembly or rearrangement of cell wall components. It means that this study allowed identifying about 350 genes encoding secreted proteins that are candidates to play roles during growth of *A. thaliana *etiolated hypocotyls. Their functional characterization will be paramount to understand cell wall architecture and assembly during an elongation process.

### Are there variations in the level of transcripts between half- and fully-grown hypocotyls?

**Figure 2:**
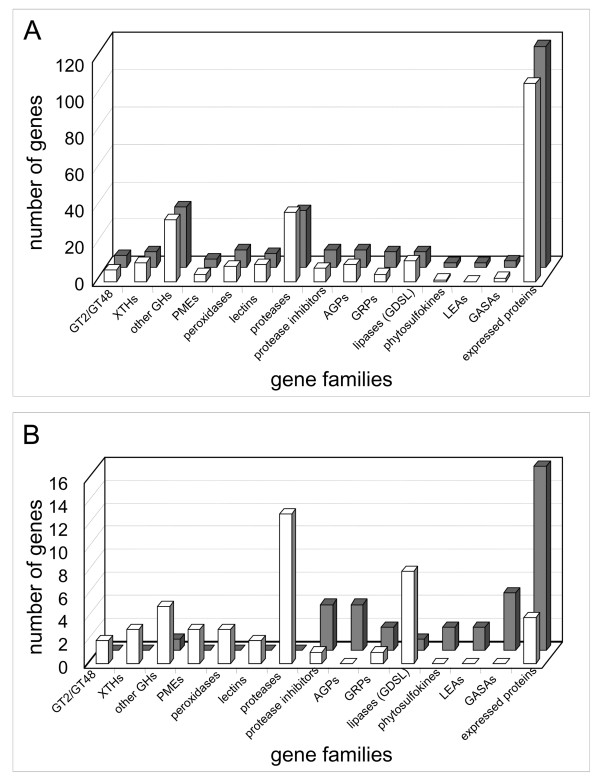
**Overview of SPGs expressed in 5- and 11-day-old hypocotyls**. **A**. Genes belonging to several families of SPGs with moderate and high level of transcripts are shown: white and grey bars are used for 5- and 11-day-old hypocotyls respectively. **B**. The genes of the same families showing significant differences in expression between 5- and 11- day-old hypocotyls are counted: genes with higher level of transcripts at 5-days are represented by white bars; genes with higher level of transcripts at 11-days are represented by grey bars. XTHs stands for xyloglucan endotransglucosylase/hydrolases, GHs for glycoside hydrolases, AGPs for arabinogalactan proteins, LEAs for late embryogenesis abundant, GASAs for gibberellic acid-stimulated *Arabidopsis *proteins, and expressed proteins for proteins with unknown function.

In 5-day-old hypocotyls, apical cells are elongating whereas basal cells are fully elongated. When compared to 11-day-old hypocotyls, where all the cells are fully elongated, the observed differences in levels of transcripts should mainly come from the cells which are at a different developmental stage, namely growing cells. Altogether, 559 genes are differentially expressed between 5- and 11-day-old hypocotyls. Among these genes, 108 encode proteins predicted to be secreted (Additional file 4). In addition, 32% of the genes having levels of transcripts modified by a factor 2 between the two developmental stages encode secreted proteins. The following detailed analysis will be focused on these genes. Sixty-three and 45 genes have a higher level of transcripts in 5- and 11-day-old hypocotyls respectively. The highest difference (8.6-fold increase) was found at 11-days for a gene encoding a glycine-rich protein (GRP, *AT2G05440*). Conversely, the largest decrease (4.5-fold) was observed at 11-days for a gene encoding a putative Asp protease (*AT5G10770*). The number of genes of selected families expressed differentially in both samples is represented in Figure 2. For comparison, the number of genes of the same families having high or moderate levels of transcripts is also represented. All the selected gene families are represented by almost the same number of genes at both developmental stages (Figure 2A). However, there are striking differences when the comparison is done with genes showing significant variation in transcript level (Figure 2B).

Several genes encoding glycosyl transferases (GTs), GHs, and PMEs have higher levels of transcripts at 5-days than at 11-days, *i.e*. at a time hypocotyls undergo active elongation. Although GTs and GHs are expected to be expressed during elongation, PMEs could play roles during both elongation and growth arrest [23]. The proportion of cells already elongated could also be significant after 5-days of growth. Alternatively, there might be a delay between synthesis of mRNAs, and production of a functional protein. This might be the case for some PMEs that are produced as polyproteins comprising an inhibitor at their N-terminus and an active enzyme at their C-terminus [23].

Other genes having higher levels of transcripts at 5-days encode peroxidases, proteases, and proteins homologous to GDSL lipases/acylhydrolases. The multiple functions of peroxidases were mentioned above. The role of proteases in cell walls during active elongation has not yet been described. It should be noted that 4 genes encoding protease inhibitors are up-regulated at 11-days, suggesting complex regulations of proteolytic activities in cell walls after the arrest of hypocotyl elongation.

Genes belonging to other gene families have higher levels of transcripts at 11-days. They code for protease inhibitors, AGPs, GRPs, late abundant embryogenesis (LEAs) proteins, phytosulfokines (PSKs), and gibberellic acid-stimulated *Arabidopsis *proteins (GASAs). AGP and FLA genes are very well-represented in the transcriptome of hypocotyls with 17 AGP genes and 13 FLA genes having detectable levels of transcripts (Additional file 1). AGPs are candidates for cell-to-cell communication [24], and FLAs were found to be associated to wood formation in poplar [15]. Concerning GRPs, they were shown to be associated to cell walls of xylem and phloem by tissue printing [25]. The great increase in the amount of transcripts of *AT2G05440 *is consistent with the development of protoxylem elements that contain GRPs [26]. Nothing is known about the role LEAs could play in fully-developed hypocotyls. PSKs were shown to promote tracheary element differentiation in *Zinnia *cell suspension cultures, and to play roles during growth [27]. Five GASA genes have higher levels of transcripts at 11-days than at 5-days. *A. thaliana GASA4 *and *GASA5 *were shown to play roles in flowering regulation and seed development [28], as well as in stem growth and flowering time [29]. Increase in the level of transcripts of the *GASA *genes in 11-day-old hypocotyls thus points at their role in elongation arrest. Finally, 16 genes encoding secreted proteins of unknown function are transcribed at higher level at 11-days. Exploring the function of these proteins will be one of the major tasks for the future.

These differences in transcript abundance between the two stages of hypocotyl growth should be taken carefully with regard to the possible functions carried out by the proteins, since many other genes from the same families are transcribed in half- and fully-grown hypocotyls, but without significant differences.

### Transcriptome vs proteome

In order to look for the consistency between levels of mRNAs and presence of the corresponding proteins in cell walls, a proteomic analysis was performed on cell walls [11], and the results were compared to those of this transcriptomic analysis. The cell wall proteomes of 5- and 11-days-old hypocotyls were achieved and a total of 137 proteins predicted to be secreted were identified (Additional file 5). When these 137 proteins were compared to the 433 SPGs with moderate and high levels of transcripts (Additional file 3), only 48 proteins matched (11.8%). Conversely, from the 228 SPGs having high levels of transcripts in etiolated hypocotyls, only 28 (12.2%) showed the corresponding proteins (Table 1). It was expected that proteomic profiling identified at least the proteins encoded by the highly-transcribed genes. The great inconsistency between the abundance of mRNAs and the presence of the corresponding proteins was surprising, but several reasons may explain this disparity. It is known that CWP extraction and identification can be challenging [18,19]. Many proteins can remain linked to the polysaccharide matrix, such as the structural proteins [30,31], or some peroxidases that might be strongly bound to pectins [32]. Others are difficult to identify because of their structure, *e.g*. highly *O*-glycosylated AGPs, which requires a special deglycosylation step [33]. Some proteins contain few linkages sensitive to tryptic digestion, and can escape identification by peptide mass mapping. Finally, low-abundant proteins elude proteomic analyses. For the proteins that were identified without particular problems such as GHs, expansins and proteases, only a few of them correspond to highly-transcribed genes. It indicates that a high level of transcripts is not always correlated with the presence of the protein in sufficient amount to be identified in proteomic approaches.

**Table 1:** Genes with high levels of transcripts in either 5- or 11-day-old hypocotyls for which the encoded proteins were identified in a proteomic study performed on the same material.

**Functional class**	**AGI number**	**Predicted or known gene function**	**5-** **days**	**11-days**	**log_2 _of ratio** **11-days/5-days**	**p-value**
**Proteins acting on carbohydrates**						
glycoside hydrolase family 16 (xyloglucan endotrans-glycosidases/hydrolases)	AT2G06850	AtXTH4	13.27	12.62	-0.65	1.96E-01
glycoside hydrolase family 20 (beta-hexosaminidase)	AT3G55260		10.40	9.90	-0.51	1.00
glycoside hydrolase family 31	AT1G68560	AtXYL1	10.97	10.57	-0.41	1.00
carbohydrate esterase family 8 (pectin methylesterase)	AT3G14310	AtPME3	11.66	10.82	-0.84	1.44E-04
alpha-expansin	AT5G02260	AtEXPA9	12.95	12.54	-0.41	1.00


**Proteases**						
cysteine protease (papain family)	AT4G01610		12.70	12.50	-0.20	1.00
aspartic protease (pepsin family)	AT3G54400		10.30	9.85	-0.45	1.00
aspartic protease (pepsin family)	AT5G10770		11.73	9.55	-2.17	0.00E+00


**Structural proteins**						
proline-rich protein (PRP)	AT1G28290		11.98	11.89	-0.09	1.00
LRR-extensin	AT3G24480	AtLRX4	10.26	10.33	0.08	1.00


**Proteins involved in oxido-reduction reactions**						
peroxidase	AT1G71695	AtPrx12	10.44	9.48	-0.96	7.29E-07
peroxidase	AT3G21770	AtPrx30	10.39	10.30	-0.08	1.00
early nodulin AtEN20 (protein homologous to blue copper binding protein)	AT4G12880	plastocyanin	11.56	11.94	0.38	1.00


**Proteins with interacting domains**						
protein homologous to lectin (curculin-like)	AT1G78850	curculin-like, mannose binding	11.87	11.37	-0.50	1.00
protein homologous to lectin (curculin-like)	AT1G78830	curculin-like, mannose binding	10.44	9.90	-0.54	1.00
protein with leucine-rich-repeat domains (LRRs)	AT3G20820	expressed protein	10.84	10.26	-0.59	1.00
enzyme inhibitor	AT1G73260	inhibitor family I3 (Kunitz-P family)	12.54	13.41	0.86	5.77E-05


**Proteins related to lipid metabolism**						
protein homologous to lipase/acylhydrolase	AT1G54030	GDSL family	10.96	10.41	-0.54	1.00


**Miscellaneous functions**						
protein homologous to phosphate-induced proteins	AT5G09440		12.68	12.14	-0.54	1.00
gibberellin regulated protein	AT5G15230	GASA4	13.16	13.93	0.77	2.75E-03
protein homologous to germin	AT1G09560	AtGLP5	11.34	11.06	-0.28	1.00
protein homologous to purple acid phosphatase	AT5G34850		12.30	11.94	-0.36	1.00
protein homologous to purple acid phosphatase	AT2G27190		11.34	11.09	-0.25	1.00


**Unknown function**						
expressed protein	AT5G11420	DUF642	12.14	12.05	-0.09	1.00
expressed protein	AT3G08030	DUF642	12.40	12.06	-0.35	1.00
expressed protein	AT4G32460	DUF 642	10.87	10.10	-0.77	2.20E-03
expressed protein	AT3G20370	MATH domain	11.42	10.92	-0.50	1.00
expressed protein	AT2G34700	Ole eI allergen domain	11.53	9.27	-2.27	0.00E+00

Genes are classified by predicted functional domains as described in Methods. The intensity of the signal is expressed as log_2_. The log_2 _of ratios between the levels of transcripts at 11-days and 5-days as well as the Bonferroni p-values are indicated.

**Figure 3:**
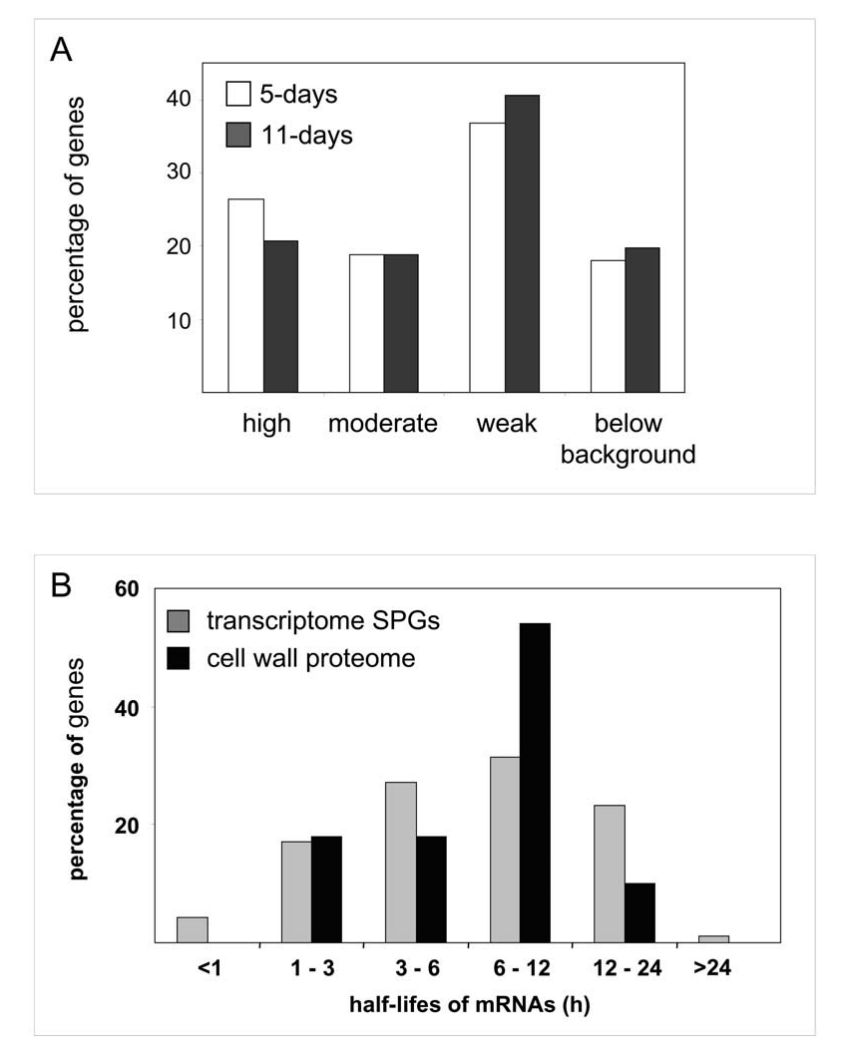
**Level of transcripts of genes encoding CWPs identified through proteomics**. **A**. The levels of transcripts of genes were determined by the CATMA analysis in 5- and 11-day-old hypocotyls (white and dark grey bars respectively). Percentage of genes falling in the following three categories are represented: high transcript level corresponds to log_2 _values of the mean signal intensity higher than 10, moderate to values between 9 and 10, and weak to values between background (6.83) and 9. **B**. Half-lifes of mRNAs (in hours) corresponding to SPGs having high or moderate levels of transcripts (grey bars) or to proteins identified through cell wall proteomics (black bars). Percentage of genes in each range of half-lifes is indicated for each set of genes. Half-lifes of mRNAs in cell suspension cultures were from [37].

In a second step, the level of transcripts of the 137 genes encoding the proteins identified through proteomics was analyzed (Figure 3A; Additional file 5). The transcript data of 31 genes was not found in the CATMA experiment since some of them have no GST or were eliminated because of poor signals of hybridization to the RNA probe. The levels of transcripts of the 106 remaining genes were surprising, for 5-days and 11-days, 36.8% and 40.6% respectively had low level of transcript while 17.9% and 19.8% respectively had levels of transcripts below background. However, all the identified proteins are assumed to be the most abundant. This suggests that the transcripts could have short half-lifes and/or that the proteins could have a low turnover. This is the case of several genes encoding proteins acting on carbohydrates (*At1 g10550*, *At2 g33160*, *At4 g18180*, *At3 g13790*, and *At4 g37950*) or oxido-reductases (*At3 g49110*, *At3 g50990*, *At4 g25980*, *At5 g64100*, *At1 g30710*, *At5 g44360*, *At5 g44410*, and *At1 g01980*). Additional experiments will be necessary to determine the half-lifes of the transcripts and of the proteins in etiolated hypocotyls.

The results obtained with the CATMA analysis were confirmed by quantitative RT-PCR (reverse transcription-polymerase chain reaction) analysis (Additional file 6). Several genes corresponding to the three cases described were chosen: high or moderate level of transcripts and proteins identified; high level of transcripts and proteins not identified; low or below background level of transcripts and proteins identified. Note that genes having very low levels of transcripts give signals below the sensitivity of the CATMA analysis.

Altogether, these results show that there is not a clear correlation between the presence of CWPs as shown by cell wall proteomic analysis and the amount of transcripts of the corresponding genes. The quality of this correlation may depend on genes and/on environmental conditions. For example, the quantification of soluble proteins of yeast at mid-log phase showed that for a given transcript level, protein levels were found to vary by more than 20-fold, whereas for a given protein level, transcript levels were found to vary 30-fold [34]. However, up-regulation of yeast genes in response to glucose or nitrogen limitation was found to be controlled at the transcriptional or post-transcriptional level respectively [35]. In *A. thaliana *and rice, changes observed in the soluble proteome in response to bacterial challenge were not strictly correlated to changes in transcript levels [36]. These results show that quantitative analysis of transcript levels is not sufficient to infer protein levels. Multilevel analysis must take into account the stability of transcripts, their availability for active translation, as well as the stability of proteins, which is certainly essential considering the high number of proteases in cell walls. With regard to transcript stability, data from a recent study aiming at measuring mRNA decay rates in *A. thaliana *cell suspension cultures [37] were used to look for half-lifes of gene transcripts identified through proteomics (Figure 3B). It can be seen that more than half of the proteins (64%) identified by cell wall proteomics correspond to genes having transcripts with rather long half-lifes (6-24 h). Conversely, no gene corresponding to proteins identified by cell wall proteomics has transcripts with half-lifes shorter than 1 h. This distribution differs from that of transcripts of genes having high or moderate level of transcripts since 48.5% of these genes have half-lifes shorter than 6 h.

As a particular case, etiolated hypocotyls of *A. thaliana *at 5-days and 11-days, 28 and 22 genes respectively showed both high level of transcripts, and encoded proteins identified by cell wall proteomics. They might be considered as good markers for cell elongation of dark-grown hypocotyls.

## Conclusion

mRNA profiling of the genes potentially involved in cell wall biogenesis (CWGs) in etiolated hypocotyls showed that more than half of them present a detectable level of transcripts. All gene families are expressed. The results suggest that both synthesis and rearrangement of wall components are required throughout hypocotyl elongation. The transcriptomic analysis of genes encoding secreted proteins showed that around 350 new genes might be implicated in this process. Understanding the biochemical and biological functions of these genes might reveal new mechanisms of cell wall expansion or of growth arrest, or new functions for the cell wall.

Around 100 genes encoding secreted proteins had significantly different levels of transcripts between growing and fully-elongated hypocotyls. As expected, genes acting on polysaccharides (GTs, GHs) had higher levels of transcripts at 5-days, whereas others encoding PMEs or peroxidases were not supposed to have higher level of transcripts during active elongation. Their function during cell elongation should be reexamined. Several genes encoding proteases also have higher level of transcripts at 5-days and could play roles in protein maturation or turn over. On the contrary, several genes encoding AGPs, protease inhibitors, and proteins homologous to gibberellin regulated proteins had higher levels of transcripts at 11-days. Their functions after the end of the elongation process remain to be found. As expected, some genes encoding GRPs were found to have much higher levels of transcripts in fully-grown hypocotyls at a time lignification is an active process. However, since all these genes belong to multigene families, one cannot rule out the fact that a similar function can be shared by several genes.

Finally, looking into the transcript level of the genes corresponding to the 137 proteins identified by proteomic analysis of the cell walls of half- and fully-grown hypocotyls, 15% were below the CATMA background. On the contrary, only 13% of the genes encoding secreted proteins with high or moderate levels of transcripts corresponded to proteins identified through proteomics. Thus, the comparison between transcript levels and presence of the corresponding proteins suggested that many genes encoding proteins secreted in cell walls are regulated at a post-transcriptional level. In conclusion, transcriptomic and proteomic data appeared to be complementary to describe the regulation of gene activity during the elongation of etiolated hypocotyls.

## Methods

### Plant material

*Arabidopsis thaliana *seedlings (ecotype Columbia 0) were grown in continuous dark in Magenta boxes on Murashige and Skoog [38] medium supplemented with 2% sucrose. Etiolated hypocotyls were collected after 5- and 11- days of culture.

### Total RNA extraction

Two RNA extractions from two biological replicates were performed for each sample (5- and 11-day-old hypocotyls). Hypocotyls were cut below the cotyledons and above the crown with sterile scissors. They were ground in liquid nitrogen in a mortar with a pestle. Extraction of total RNAs was performed using the SV Total RNA Isolation kit according to manufacturer's instructions (Promega France, Charbonnières, France). For each RNA extraction, 750 mg of ground hypocotyls were used. Typically, about 110 μg of total RNAs were obtained.

### Transcriptome studies

Microarray analysis was carried out at the Unité de Recherche en Génomique Végétale (Evry, France), using the CATMA array [10,39], containing 24,576 GSTs from *A. thaliana*. RNA samples from the two independent biological replicates were isolated and separately analyzed. For each comparison, one technical replication with fluorochrome reversal was performed for each RNA sample (*i.e*. four hybridizations in two dye swaps per comparison). The reverse transcription of RNA in the presence of Cy3-dUTP or Cy5-dUTP (Perkin-Elmer-NEN Life Science Products), the hybridization of labeled samples to the slides, and the scanning of the slides were performed as described in Lurin *et al *[40].

### Statistical analysis of microarray data

Statistical analysis was based on two dye swaps (*i.e*. four arrays, each containing 24,576 gene-specific tags and 384 controls) as described in Gagnot *et al *(2007). To estimate the transcript level of each gene, a background value was obtained by addition of the average background value to 2 background standard deviations. The average background value was calculated using a subset of 1000 non-expressed genes found in the whole CATMA database . The background value was not subtracted from the data presented in this paper, but was considered for the interpretation of the results. To determine differentially expressed genes, we performed a paired *t*-test on the log ratios, assuming that the variance of the log ratios was the same for all genes. Spots displaying extreme variance (too small or too large) were excluded. The raw *P*-values were adjusted by the Bonferroni method, which controls the Family Wise Error Rate. We considered as being differentially expressed the genes with a Bonferroni *P*-value ≤ 0.05, as described in [41]. We use the Bonferroni method (with a type I error equal to 5%) in order to keep a strong control of the false positives in a multiple-comparison context [42].

### Data deposition

Microarray data from this article were deposited at Gene Expression Omnibus ; accession No. GSE14648) and at CATdb ( Project RS05-11_Hypocotyls) according to the "Minimum Information About a Microarray Experiment" standards.

### RT-PCR

cDNA first strands were obtained from total RNAs using 1 μg total RNAs and SuperScript™ II reverse transcriptase (Invitrogen, Carlsbad, San Diego, CA, USA). As a control, the same amount of pig desmin RNA was added in each sample. Quantitative PCR was performed using a Roche lightcycler system (Roche Diagnostics, Meylan, France) according to manufacturer's recommendations. The sequences of oligonucleotide primers used for amplification is provided in Additional file 7. Using the results from quantitative PCR to determine the number of amplification cycles required to be in a linear range for all genes of interest, semi-quantitative PCR was performed (Additional file 6). The amplified fragments were analyzed by electrophoresis in polyacrylamide gels in standardized conditions. In each case, presence of a fragment of the expected size was checked after staining with ethidium bromide.

### Bioinformatic analyses

Sub-cellular localization and length of signal peptides were predicted using PSORT  and TargetP [43,44]. Prediction of transmembrane domains was done with Aramemnon [45]. Molecular masses and pI values were calculated using the aBi program . Homologies to other proteins were searched for using BLAST programs [46]. Identification of protein families and functional domains was performed using MyHits  and InterProScan [47]. TargetP, Aramemnon, and InterProScan software were combined to provide the *ProtAnnDB *friendly user web interface [48]. A MySQL (v4.1) database and PHP5 scripts were used to store, order and extract numeric and qualitative data

All the protein families chosen in our CWG list were annotated by experts. GHs and CEs were classified according to the CAZy database [49] at the Cell Wall Genomics website . The GT77 family was annotated according to Egelund *et al *[50]. XTHs and expansins were named according to  and  respectively. AGPs and FLAs were named according to Schultz *et al *[51], Johnson *et al *[52], Van Hengels and Roberts [53], and Liu and Mehdy [54]. Proteins homologous to COBRA, LRXs and Hyp/Pro-rich proteins were annotated according to Roudier *et al *[16], Baumberger *et al *[55], and Fowler *et al *[56] respectively. The lignin toolbox was proposed by Raes *et al *[57]. Peroxidases were named as in the PeroxiBase [58]. Laccases were annotated as in Pourcel *et al *[59] and McCaig *et al *[60]. SKU-like proteins and phytocyanins were described in Jacobs and Roe [61], and Nersissian and Shipp [62] respectively. Subtilases are listed at . Pectin methylesterase inhibitors were annotated by Dr J Pelloux (University of Amiens, France).

## Authors' contributions

EJ conceived the study, participated in its design, coordination, analysis of data, and drafted the manuscript. DR and MI carried out the culture of plants, growth curve determination, RNA extraction and PCR-analysis. HSC was involved in bioinformatic analyses. LS-T and J-PR performed the microarray and statistical analyses of the results. RP-L contributed to the analysis of data and to drafting of the manuscript. All authors read and approved the final manuscript.

## Supplementary Material

Additional file 1 **Cell Wall Genes (CWGs) with detectable levels of transcripts in 5- or 11-day-old hypocotyls**. The data provided represent the CWGs with their level of transcripts and statistical analysis. Click here for file

Additional file 2 **Cell Wall Genes (CWGs) with high levels of transcripts in either 5- or 11-day-old hypocotyls**. The data provided represent the CWGs having high levels of transcripts. Click here for file

Additional file 3 **Secretory Pathway Genes (SPGs) with moderate or high levels of transcripts in 5- and 11-day-old etiolated hypocotyls**. The data provided represent the levels of transcripts of SPGs and the corresponding statistical analyses. Click here for file

Additional file 4 **Secretory Pathway Genes (SPGs) with modulated levels of transcripts in 5- and 11-day-old etiolated hypocotyls**. The data provided represent the transcript levels of SPGs differentially expressed between the two samples. Click here for file

Additional file 5 **Proteins extracted and identified by mass spectrometry from purified cell walls of 5- and 11-day-old etiolated hypocotyls of *A. thaliana***. The data provided represent the levels of transcripts of the genes encoding the proteins identified by proteomics. Click here for file

Additional file 6 **RT-PCR analysis of the transcripts of some genes encoding CWPs identified through proteomics**. The data provided represent the controls of microarray data by quantitative RT-PCR for some selected genes. Click here for file

Additional file 7 **Nucleotide primers used for PCR amplifications**. The sequences of the oligonucleotide primers used for PCR analysis are listed in this file. Click here for file
