# Supplementary material for: Cell wall biogenesis of Arabidopsis thaliana elongating cells: transcriptomics complements proteomics
Source: BMC Genomics. 2009 Oct 31;10:505. doi: 10.1186/1471-2164-10-505 (PMC2774874; doi:10.1186/1471-2164-10-505)
Supplement: Additional file 1 — Cell Wall Genes (CWGs) with detectable levels of transcripts in 5- or 11-day-old hypocotyls. The data provided represent the CWGs with their level of transcripts and statistical analysis. [file 1471-2164-10-505-S1.pdf]

Additional file 1. Cell Wall Genes (CWGs) with detectable levels of transcripts in 5- and 11-day-old hypocotyls. CWGs were selected according to several papers and databases as mentioned at the bottom of the Table. The intensity of the signal is expressed as log_2, the ratio between the levels of transcripts at 11 days and 5 days as well as the p-value are indicated. CWGs are classified by gene family and level of transcripts: below background in black, low level in blue (between background and 9), moderate level in green (between 9 and 10) and high level in red (higher than 10). Significant Bonferroni p-values are highlighted in green (p<5%)

Gene family	AGI number	Predicted or known gene function	CATMA probe	5 day-old hypocotyls	11 day-old hypocotyls	log_2 of ratio 11days/5 days	p-value
prolyl-4-hydroxylases [a]	AT5G18900		CATMA5A17270	10.24	10.29	0.05	1.00
	AT2G43080	AtP4H1	CATMA2A41480	9.18	9.28	0.10	1.00
	AT4G33910		CATMA4A35710	9.72	9.42	-0.30	1.00
	AT1G20270		CATMA1A19270	7.99	8.05	0.05	1.00
	AT3G28480		CATMA3A28370	7.79	7.62	-0.17	1.00
	AT4G25600		CATMA4A27290	7.23	7.27	0.04	1.00
nucleotide-sugar interconversion pathway [b]	AT5G39320	UGD1	CATMA5A34920	11.58	10.81	-0.77	2.19E-03
	AT1G08200	AXS2	CATMA1A07190	10.71	10.61	-0.10	1.00
	AT5G15490	UGD3	CATMA5A13740	10.50	10.00	-0.51	1.00
	AT5G28840	GME1	CATMA5A26920	9.89	9.75	-0.14	1.00
	AT1G53500	RHM2	CATMA1A44530	9.77	9.49	-0.27	1.00
	AT1G17890	GER2	CATMA1A16900	9.73	9.45	-0.28	1.00
	AT3G46440	SUD1	CATMA3A39510	9.33	9.03	-0.30	1.00
	AT4G30440	GAE1	CATMA4A32050	9.29	8.66	-0.63	0.29
	AT3G23820	GAE6	CATMA3A23780	9.25	8.73	-0.52	1.00
	AT2G27860	AXS1	CATMA2A26260	8.95	8.98	0.03	1.00
	AT4G00110	GAE3	CATMA4A00110	8.63	8.48	-0.16	1.00
	AT1G30620	UXE1 (MUR4)	CATMA1A28670	8.29	8.18	-0.11	1.00
	AT1G73250	GER1	CATMA1A62530	8.14	8.22	0.08	1.00
	AT1G02000	GAE2	CATMA1A00990	8.12	8.08	-0.04	1.00
	AT5G66280	GMD1	CATMA5A61645	8.06	7.82	-0.24	1.00
	AT2G47650	AUD2	CATMA2A46090	8.05	7.72	-0.33	1.00
	AT1G64440	UGE4	CATMA1A53740	7.97	7.84	-0.13	1.00
	AT1G12780	UGE1	CATMA1A11765	7.73	8.13	0.40	1.00
	AT1G26570	UGD4	CATMA1A24800	7.72	7.40	-0.32	1.00
	AT2G34850	UXE2	CATMA2A32970	7.55	7.55	-0.01	1.00
	AT3G62830	AUD	CATMA3A55995	7.55	7.32	-0.23	1.00
	AT2G45310	GAE4	CATMA2A43710	7.49	7.44	-0.05	1.00
	AT1G63000	UER1	CATMA1A52170	7.38	7.13	-0.25	1.00
	AT4G23920	UGE2	CATMA4A25693	7.33	7.41	0.08	1.00
	AT3G51160	GMD2 (MUR1)	CATMA3A44165	6.88	6.91	0.03	1.00
glycosyl transferases (GTs)							
GT8 [c]	AT1G77130	Group A	CATMA1A66340	10.11	9.79	-0.32	1.00
	AT5G18480	Group E	CATMA5A16780	9.77	9.48	-0.29	1.00
	AT2G20810	Group D (GAUT10)	CATMA2A19380	9.28	8.95	-0.33	1.00
	AT3G62660	Group C (GATL7)	CATMA3A55830	9.14	8.75	-0.38	1.00
	AT3G02350	Group D (GAUT9)	CATMA3A01330	9.08	8.76	-0.32	1.00
	AT1G19300	Group C (GATL1=Parvus)	CATMA1A18330	8.74	8.59	-0.15	1.00
	AT1G24170	Group C (GATL8)	CATMA1A23060	8.55	8.20	-0.35	1.00
	AT3G61130	Group D (GAUT1)	CATMA3A54300	8.41	8.30	-0.12	1.00
	AT3G01040	Group D (GAUT13)	CATMA3A00060	8.21	8.10	-0.11	1.00
	AT5G15470	Group D (GAUT14)	CATMA5A13720	8.12	7.95	-0.17	1.00
	AT2G38650	Group D (GAUT7)	CATMA2A36930	8.10	7.99	-0.11	1.00
	AT1G13250	Group C (GATL3)	CATMA1A12260	8.07	7.73	-0.35	1.00
	AT4G38270	Group D (GAUT3)	CATMA4A39780	8.00	8.29	0.28	1.00
	AT1G18580	Group D (GAUT11)	CATMA1A17620	7.99	7.72	-0.26	1.00
	AT3G18660	Group A (PGS1P1)	CATMA3A18270	7.89	7.48	-0.41	1.00
	AT5G47780	Group D (GAUT4)	CATMA5A43750	7.70	7.63	-0.07	1.00
	AT1G70090	Group C (GATL9)	CATMA1A59370	7.60	7.37	-0.23	1.00
	AT3G28340	Group C (GATL10)	CATMA3A28190	7.59	7.49	-0.10	1.00
	AT1G60450	Group B	CATMA1A49460	7.25	7.01	-0.24	1.00
	AT1G02720	Group C (GATL5)	CATMA1A01680	7.19	6.98	-0.21	1.00
	AT3G06260	Group C (GATL4)	CATMA3A05330	7.17	7.34	0.18	1.00
	AT1G08990	Group A	CATMA1A07830	7.05	6.87	-0.18	1.00
	AT3G58790	Group D (GAUT15)	CATMA3A51790	6.98	7.00	0.03	1.00
	AT1G56600	Group B (AtGolS2)	CATMA1A47690	6.94	7.45	0.51	1.00
GT31 [d]	AT4G00300	Group A	CATMA4A00350	10.06	9.55	-0.51	1.00
	AT1G05170	Group E	CATMA1A04030	9.52	9.07	-0.45	1.00
	AT3G14960	Group C	CATMA3A14310	9.03	9.27	0.24	1.00
	AT1G77810	Group E	CATMA1A66970	8.64	8.67	0.04	1.00
	AT2G32430	Group E	CATMA2A30720	8.42	7.88	-0.54	1.00
	AT3G11420	Group A	CATMA3A10410	8.18	8.39	0.21	1.00
	AT2G26100	Group C	CATMA2A24460	7.97	7.83	-0.13	1.00
	AT1G53290	Group C	CATMA1A44310	7.63	7.61	-0.02	1.00
	AT3G06440	Group B	CATMA3A05560	7.45	7.37	-0.09	1.00
	AT5G41460	Group A	CATMA5A37060	7.33	6.85	-0.48	1.00
	AT1G26810	Group B	CATMA1A25030	7.29	7.16	-0.13	1.00
	AT5G62620	Group B	CATMA5A58200	7.14	7.10	-0.04	1.00
	AT1G33250	Group A	CATMA1A31530	7.12	7.19	0.07	1.00
	AT4G26940	Group F	CATMA4A28520	7.11	7.27	0.15	1.00
	AT5G53340	Group D	CATMA5A49250	7.06	7.10	0.05	1.00
	AT1G32930	Group E	CATMA1A31230	6.89	6.79	-0.09	1.00
	AT2G25300	Group D	CATMA2A23620	6.88	6.85	-0.03	1.00
	AT1G27120	Group B	CATMA1A25350	6.82	6.86	0.04	1.00
GT34 [e]	AT2G22900		CATMA2A21380	10.26	9.71	-0.55	1.00
	AT4G02500		CATMA4A02820	9.70	9.40	-0.30	1.00
	AT3G62720	XT1	CATMA3A55880	8.01	8.02	0.01	1.00
	AT1G74380		CATMA1A63770	8.00	7.64	-0.36	1.00
	AT1G18690		CATMA1A17720	6.75	6.86	0.11	1.00
GT37 [f]	AT1G14070	FUT7	CATMA1A13020	7.40	7.31	-0.09	1.00
GT47 [g]	AT2G28110	Group E (FRA8)	CATMA2A26510	11.69	11.26	-0.43	1.00
	AT5G22940	Group E	CATMA5A20440	9.53	9.19	-0.34	1.00
	AT5G61840	Group E (AtGUT2)	CATMA5A57440	9.28	9.14	-0.13	1.00
	AT4G38040	Group D	CATMA4A39540	8.62	8.48	-0.14	1.00
	AT4G16745	Group D	CATMA4A17710	8.38	8.35	-0.02	1.00
	AT1G27440	Group E (AtGUT1)	CATMA1A25670	8.24	8.07	-0.18	1.00
	AT2G20370	Group A (MUR3)	CATMA2A18850	8.16	7.93	-0.23	1.00
	AT4G22580	Group A (AtGT19)	CATMA4A24310	8.10	7.89	-0.22	1.00
	AT1G21480	Group D	CATMA1A20530	7.94	7.82	-0.11	1.00
	AT3G57630	Group E	CATMA3A50640	7.93	7.64	-0.29	1.00
	AT5G33290	Group C	CATMA5A28900	7.90	7.78	-0.12	1.00
	AT2G31990	Group A (AtGT15)	CATMA2A30260	7.80	7.81	0.00	1.00
	AT1G67410	Group B	CATMA1A56730	7.72	7.64	-0.08	1.00
	AT2G35100	Group B (ARAD1)	CATMA2A33240	7.71	7.52	-0.19	1.00
	AT5G62220	Group A (AtGT18)	CATMA5A57810	7.56	7.40	-0.15	1.00
	AT1G34270	Group B	CATMA1A32570	7.46	7.32	-0.14	1.00
	AT5G16890	Group B	CATMA5A15200	7.45	7.34	-0.11	1.00
	AT1G74680	Group B	CATMA1A64050	7.26	7.16	-0.10	1.00
	AT5G44930	Group B	CATMA5A40770	7.21	7.33	0.12	1.00
	AT5G19670	Group D	CATMA5A18100	7.00	6.93	-0.07	1.00
	AT5G11610	Group D	CATMA5A10380	6.98	6.92	-0.06	1.00
	AT2G29040	Group A (AtGT11)	CATMA2A27430	6.97	6.82	-0.15	1.00
	AT4G13990	Group A (AtGT14)	CATMA4A14190	6.95	6.97	0.02	1.00
	AT1G68470	Group A (AtGT17)	CATMA1A57850	6.93	6.87	-0.06	1.00
	AT5G25820	Group D	CATMA5A23490	6.91	6.84	-0.07	1.00

GT77 [h]	AT1G19360		CATMA1A18380	10.37	10.07	-0.29	1.00
	AT2G35610		CATMA2A33740	8.13	7.99	-0.14	1.00
	AT5G44820		CATMA5A40660	7.49	7.31	-0.18	1.00
	AT1G14590		CATMA1A13610	7.40	7.26	-0.13	1.00
	AT1G75110	RRA2	CATMA1A64440	7.05	6.97	-0.08	1.00
	AT1G70630		CATMA1A59910	6.79	6.92	0.13	1.00
GT2 (cellulose synthases) [i]	AT5G09870	AtCESA5 (unknown function)	CATMA5A08637	10.43	9.65	-0.78	1.64E-03
	AT5G05170	AtCESA3 (primary wall)	CATMA5A04375	10.40	9.88	-0.52	1.00
	AT4G39350	AtCESA2 (unknown function)	CATMA4A40743	10.23	9.52	-0.72	1.78E-02
	AT4G32410	AtCESA1 (primary wall)	CATMA4A34150	10.05	9.53	-0.52	1.00
	AT5G44030	AtCesA4 (secondary wall)	CATMA5A39800	7.93	7.64	-0.29	1.00
	AT2G25540	AtCesA10 (unknown function)	CATMA2A23880	7.57	7.43	-0.14	1.00
	AT5G17420	AtCesA7 (secondary wall, AtIRX3))	CATMA5A15680	7.12	6.88	-0.23	1.00
GT2 (cellulose synthases-like) [i]	AT5G03760	AtCSLA9	CATMA5A02940	9.86	9.22	-0.64	0.20
	AT3G28180	AtCSLC4	CATMA3A06563	9.54	9.36	-0.17	1.00
	AT3G07330	AtCSLC6	CATMA3A28010	8.87	9.22	0.35	1.00
	AT2G35650	AtCSlA7	CATMA2A33860	8.70	8.59	-0.11	1.00
	AT5G22740	AtCSLA2	CATMA5A20200	8.66	8.27	-0.39	1.00
	AT1G23480	AtCslA3	CATMA1A22512	7.40	7.47	0.06	1.00
	AT1G55850	AtCSLE1	CATMA1A47044	7.38	7.81	0.43	1.00
	AT5G16190	CslA11	CATMA5A14470	7.08	7.14	0.06	1.00
	AT2G33100	AtCslD1	CATMA2A31270	7.06	7.03	-0.03	1.00
	AT4G07960	CslC12	CATMA4A07460	6.88	6.81	-0.07	1.00
GT48 (callose synthases) [j]	AT1G05570	AtGSL6 (CALS1)	CATMA1A04500	9.77	9.06	-0.72	1.84E-02
	AT2G31960	AtGSL3	CATMA2A30240	9.51	9.25	-0.26	1.00
	AT5G13000	AtGSL12	CATMA5A11220	8.94	8.81	-0.13	1.00
	AT3G07160	AtGSL10	CATMA3A06350	8.50	8.38	-0.12	1.00
	AT2G36850	AtGSL8	CATMA2A35110	8.40	8.40	0.00	1.00
vesicle trafficking (emp24/gp25L/p24 family protein) [k]	AT1G69460		CATMA1A58750	10.88	10.55	0.33	1.00
	AT1G57620		CATMA1A47920	9.58	9.33	0.25	1.00
	AT3G07680		CATMA3A06940	9.16	8.90	0.26	1.00
	AT1G14010		CATMA1A12960	8.60	8.42	0.18	1.00
	AT1G21900		CATMA1A21000	8.23	8.02	0.21	1.00
	AT1G09580		CATMA1A08430	7.27	7.43	-0.16	1.00
glycosyl hydrolases (GHs)							
GH9 (endoglucanases) [l]	AT4G02290		CATMA4A02560	11.10	10.08	-1.02	1.57E-07
	AT1G64390		CATMA1A53700	10.68	10.19	-0.49	1.00
	AT5G49720	AtKOR	CATMA5A45658	9.64	9.94	0.30	1.00
	AT1G70710	AtCEL1	CATMA1A59990	9.32	8.93	-0.39	1.00
	AT1G75680		CATMA1A64980	8.37	8.20	-0.17	1.00
	AT4G24260		CATMA4A25995	7.70	7.88	0.18	1.00
	AT1G71380		CATMA1A60700	7.70	7.68	-0.02	1.00
	AT2G32990		CATMA2A31180	7.35	7.23	-0.12	1.00
	AT4G39000		CATMA4A40450	6.68	6.83	0.15	1.00
GH16 (xyloglucan endotransglycosylases/hydrolases) (XTHs) [m]	AT4G14130	At-XTH15	CATMA4A14375	13.61	12.31	-1.31	0.00E+00
	AT2G06850	At-XTH4	CATMA2A05540	13.27	12.62	-0.65	1.96E-01
	AT4G30290	At-XTH19	CATMA4A31900	12.37	12.15	-0.22	1.00
	AT1G32170	At-XTH30	CATMA1A30510	11.24	11.17	-0.06	1.00
	AT1G11545	At-XTH8	CATMA1A10510	10.48	10.34	-0.14	1.00
	AT2G01850	At-XTH27	CATMA2A00905	10.42	10.34	-0.08	1.00
	AT3G44990	At-XTH31	CATMA3A38015	10.10	8.76	-1.35	0.00E+00
	AT4G37800	At-XTH7	CATMA4A39300	9.89	8.65	-1.24	0.00E+00
	AT4G30270	At-XTH24 (MERI-5)	CATMA4A31885	9.88	10.14	0.25	1.00
	AT1G14720	At-XTH28	CATMA1A13765	9.72	9.73	0.00	1.00
	AT5G13870	At-XTH5	CATMA5A12105	9.02	8.63	-0.39	1.00
	AT2G36870	At-XTH32	CATMA2A35140	8.82	7.91	-0.91	6.19E-06
	AT3G23730	At-XTH16	CATMA3A23670	8.17	7.89	-0.29	1.00
	AT5G48070	At-XTH20	CATMA5A44050	7.93	8.12	0.19	1.00
	AT4G25820	At-XTH14	CATMA4A27496	7.47	7.43	-0.04	1.00
	AT2G14620	At-XTH10	CATMA2A13330	7.07	7.08	0.01	1.00
GH17 [n]	AT5G42100	Group C	CATMA5A37820	10.36	10.13	-0.22	1.00
	AT3G04010	Group B	CATMA3A02980	10.25	9.24	-1.01	6.09E-08
	AT5G58090	Group B	CATMA5A53850	9.83	10.16	0.33	1.00
	AT5G42720	Group C	CATMA5A38510	9.45	9.08	-0.37	1.00
	AT3G07320	Group E	CATMA3A06560	9.33	8.76	-0.57	1.00
	AT5G20330	Group A (AtBG4)	CATMA5A18800	9.07	9.09	0.02	1.00
	AT4G31140	Group B	CATMA4A32830	8.79	8.43	-0.36	1.00
	AT2G01630	Group D	CATMA2A00680	8.61	8.40	-0.21	1.00
	AT2G26600	Group C	CATMA2A24920	8.59	8.51	-0.08	1.00
	AT5G58480	Group B	CATMA5A54240	8.23	7.75	-0.47	1.00
	AT3G55430	Group C	CATMA3A48400	7.89	7.78	-0.12	1.00
	AT4G29360	Group D	CATMA4A30990	7.84	7.56	-0.28	1.00
	AT5G20870	Group B	CATMA5A19400	7.68	7.46	-0.22	1.00
	AT5G56590	Group D	CATMA5A52400	7.66	7.54	-0.13	1.00
	AT1G66250	Group D	CATMA1A55520	7.54	7.49	-0.06	1.00
	AT2G19440	Group B	CATMA2A18020	7.51	7.46	-0.04	1.00
	AT4G17180	Group B	CATMA4A18200	7.50	7.38	-0.13	1.00
	AT1G30080	Group C	CATMA1A28100	7.38	7.34	-0.04	1.00
	AT1G64760	Group B	CATMA1A54070	7.36	7.27	-0.08	1.00
	AT2G27500	Group C	CATMA2A25930	7.28	7.49	0.21	1.00
	AT4G16260	Group A	CATMA4A17080	7.24	7.30	0.05	1.00
	AT1G32860	Group C	CATMA1A31160	7.14	7.16	0.02	1.00
	AT5G18220	Group B	CATMA5A16490	7.12	7.02	-0.11	1.00
	AT3G15800	Group C	CATMA3A15190	7.11	6.96	-0.15	1.00
	AT3G61810	Group E	CATMA3A54970	7.04	6.92	-0.11	1.00
	AT5G64790	Group B	CATMA5A60230	6.89	6.68	-0.20	1.00
	AT5G20560	Group A	CATMA5A19060	6.88	6.91	0.03	1.00
	AT4G18340	Group C	CATMA4A19400	6.85	6.79	-0.06	1.00
GH18 (yieldins) [o]	AT5G24090		CATMA5A21620	7.73	7.63	-0.10	1.00
	AT4G19810		CATMA4A21020	7.19	6.84	-0.35	1.00
	AT4G19750		CATMA4A20980	6.86	7.00	0.14	1.00
	AT4G19730		CATMA4A20960	6.84	6.81	-0.03	1.00
GH28 (polygalacturonases) [p]	AT4G23820	Group A	CATMA4A25640	10.23	9.89	-0.34	1.00
	AT3G06770	Group A	CATMA3A05990	9.51	9.32	-0.19	1.00
	AT3G61490	Group A	CATMA3A54630	8.91	8.80	-0.11	1.00
	AT5G41870	Group A	CATMA5A37590	8.78	8.46	-0.32	1.00
	AT3G16850	Group A	CATMA3A16240	8.39	8.18	-0.20	1.00
	AT3G62110	Group A	CATMA3A55250	8.36	8.34	-0.02	1.00
	AT1G80170	Group D	CATMA1A69350	7.91	7.91	0.00	1.00
	AT3G15720	Group B	CATMA3A15120	7.83	8.35	0.52	1.00
	AT3G55790	Group A	CATMA3A48760	7.83	8.10	0.27	1.00
	AT5G49215	Group A	CATMA5A45170	7.80	7.79	-0.01	1.00
	AT3G42950	Group A	CATMA3A35240	7.74	7.61	-0.13	1.00
	AT4G23500	Group A	CATMA4A25330	7.35	7.13	-0.22	1.00
	AT1G70500	Group D	CATMA1A59770	7.18	7.15	-0.02	1.00
	AT2G43870	Group G	CATMA2A42280	7.03	6.91	-0.12	1.00
	AT1G19170	Group A	CATMA1A18210	6.92	6.90	-0.03	1.00
	AT5G14650	Group C	CATMA5A12860	6.86	6.84	-0.03	1.00
	AT1G60590	Group C	CATMA1A49600	6.68	6.91	0.23	1.00
GH35 (beta-galactosidases) [q]	AT5G56870	AtBGAL4	CATMA5A52625	13.37	12.75	-0.62	4.79E-01
	AT1G45130	AtBGAL5	CATMA1A37985	11.77	11.16	-0.61	5.91E-01
	AT5G20710	AtBGAL7	CATMA5A19240	9.76	9.09	-0.68	7.12E-02
	AT5G63810	AtBGAL10	CATMA5A59336	8.86	8.53	-0.33	1.00
	AT3G52840	AtBGAL2	CATMA3A45773	8.64	8.26	-0.38	1.00
	AT5G63800	AtBGAL6	CATMA5A59333	7.97	7.50	-0.47	1.00
	AT2G28470	AtBGAL8	CATMA2A26900	7.39	7.28	-0.11	1.00
	AT1G72990	AtBGAL17	CATMA1A62200	7.29	7.31	0.02	1.00
	AT3G13750	AtBGAL1	CATMA3A12935	7.13	7.07	-0.06	1.00

carbohydrate esterases (CEs)							
CE8 (pectin methylesterases) [r]	AT2G43050		CATMA2A41450	12.72	11.24	-1.48	0.00E+00
	AT3G49220		CATMA3A42250	11.84	11.15	-0.69	3.94E-02
	AT3G14310	AtPME3	CATMA3A13580	11.66	10.82	-0.84	1.44E-04
	AT1G53840	AtPME1	CATMA1A44933	9.72	9.75	0.03	1.00
	AT3G43270		CATMA3A35650	9.30	8.89	-0.41	1.00
	AT1G53830	AtPME2	CATMA1A44930	9.01	8.39	-0.62	5.17E-01
	AT4G33220		CATMA4A34970	8.59	8.11	-0.48	1.00
	AT2G26440		CATMA2A24760	8.27	7.68	-0.59	1.00
	AT5G09760		CATMA5A08570	8.23	8.00	-0.23	1.00
	AT3G29090		CATMA3A29170	8.16	8.55	0.39	1.00
	AT5G64640		CATMA5A60060	7.87	8.27	0.39	1.00
	AT3G10720		CATMA3A09760	7.77	7.57	-0.19	1.00
	AT1G05310		CATMA1A04190	7.66	7.40	-0.26	1.00
	AT5G47500		CATMA5A43480	7.53	7.33	-0.20	1.00
	AT4G02330		CATMA4A02630	7.46	7.26	-0.20	1.00
	AT5G53370		CATMA5A49270	7.30	7.03	-0.27	1.00
	AT3G05610		CATMA3A04630	7.26	7.31	0.05	1.00
	AT3G59010		CATMA3A52040	7.25	6.90	-0.35	1.00
CE13 (pectin acylesterases) [s]	AT3G05910		CATMA3A04940	10.55	10.41	-0.15	1.00
	AT4G19410		CATMA4A20590	9.23	8.97	-0.26	1.00
	AT2G46930		CATMA2A45390	8.04	7.83	-0.21	1.00
	AT3G09410		CATMA3A08300	7.61	7.42	-0.19	1.00
	AT3G62060		CATMA3A55190	7.63	7.45	-0.18	1.00
polysaccharide lyases (PLs)							
PL1 (pectate lyases) [t]	AT5G48900	Group A	CATMA5A44810	11.58	10.93	-0.65	1.52E-01
	AT3G07010	Group A	CATMA3A06210	11.30	10.91	-0.39	1.00E+00
	AT1G04680	Group A	CATMA1A03520	11.30	10.97	-0.33	1.00
	AT1G67750	Group A	CATMA1A57110	10.89	10.35	-0.54	1.00
	AT4G24780	Group A	CATMA4A26470	9.69	10.36	0.67	9.00E-02
	AT5G63180	Group A	CATMA5A58780	8.86	8.44	-0.42	1.00
	AT3G55250	Group B	CATMA3A48250	8.53	8.40	-0.13	1.00
	AT3G09540	Group B	CATMA3A08400	8.31	7.78	-0.53	1.00
	AT3G55140	Group B	CATMA3A48150	7.73	7.82	0.09	1.00
	AT4G13710	Group A	CATMA4A13890	7.73	7.47	-0.26	1.00
	AT5G04310	Group C	CATMA5A03510	7.50	7.44	-0.06	1.00
	AT3G24670	Group A	CATMA3A24620	7.43	7.14	-0.29	1.00
	AT3G27400	Group A	CATMA3A27260	6.86	7.17	0.30	1.00
PL4 (rhamnogalacturonan lyases) [u]	AT1G09910		CATMA1A08780	8.22	8.28	-0.06	1.00
	AT4G24430		CATMA4A26170	7.22	7.06	0.17	1.00
expansins [v]	AT5G02260	AtEXPA9 (alpha expansin)	CATMA5A01340	12.95	12.54	-0.41	1.00
	AT1G69530	AtEXPA1 (alpha expansin)	CATMA1A58850	11.10	11.63	0.52	1.00
	AT2G40610	AtEXPA8 (alpha expansin)	CATMA2A38930	10.49	10.40	-0.09	1.00
	AT4G28250	AtEXPB3 (beta expansin)	CATMA4A29890	10.01	10.66	0.65	1.97E-01
	AT2G28950	AtEXPA6 (alpha expansin)	CATMA2B27340	9.73	9.81	0.08	1.00
	AT1G20190	AtEXPA11 (alpha expansin)	CATMA1A19190	9.56	9.31	-0.24	1.00
	AT2G37640	AtEXPA3 (alpha expansin)	CATMA2A35920	8.81	8.94	0.13	1.00
	AT2G20750	AtEXPB1 (beta expansin)	CATMA2A19310	8.72	8.50	-0.22	1.00
	AT2G03090	AtEXPA15 (alpha expansin)	CATMA2A02000	8.66	8.66	-0.01	1.00
	AT4G38210	AtEXPA20 (alpha expansin)	CATMA4A39720	8.58	8.60	0.02	1.00
	AT4G38400	AtEXLA2 (expansin like family A)	CATMA4A39920	8.32	8.09	-0.23	1.00
	AT1G26770	AtEXPA10 (alpha expansin)	CATMA1A24995	8.18	8.38	0.20	1.00
	AT3G55500	AtEXPA16 (alpha expansin)	CATMA3A48450	7.79	7.75	-0.04	1.00
	AT3G45960	AtEXLA3 (expansin like family A)	CATMA3A38990	7.67	7.64	-0.03	1.00
	AT5G56320	AtEXPA14 (alpha expansin)	CATMA5A52120	7.47	7.15	-0.32	1.00
	AT5G05290	AtEXPA2 (alpha expansin)	CATMA5A04475	7.28	7.51	0.23	1.00
	AT3G15370	AtEXPA12 (alpha expansin)	CATMA3A14790	7.06	7.44	0.38	1.00
	AT4G17030	AtEXLB1 (expansin like family B)	CATMA4A18050	6.80	6.94	0.14	1.00
arabinogalactan proteins (AGPs) [w]	AT2G14890	AtAGP9	CATMA2A13650	12.43	12.55	0.12	1.00
	AT5G11740	AtAGP15 (AG peptide)	CATMA5A10540	12.04	12.70	0.66	1.26E-01
	AT1G28290	AtAGP31	CATMA1A26460	11.98	11.89	-0.09	1.00
	AT3G13520	AtAGP12 (AG peptide)	CATMA3A12660	11.93	12.97	1.04	1.23E-08
	AT5G10430	AtAGP4	CATMA5A09183	11.76	11.86	0.10	1.00
	AT5G53250	AtAGP22 (AG peptide)	CATMA5A49170	9.98	10.94	0.96	0.00
	AT3G61640	AtAGP20 (AG peptide)	CATMA3A54770	9.91	10.65	0.74	6.96E-03
	AT5G64310	AtAGP1	CATMA5A59763	9.62	9.86	0.24	1.00
	AT2G47930	AtAGP26	CATMA2A46360	9.07	9.62	0.55	1.00
	AT5G18690	AtAGP25	CATMA5A17040	8.68	8.80	0.12	1.00
	AT4G37450	AtAGP18 (Lys-rich AGP)	CATMA4A39020	8.64	8.41	-0.22	1.00
	AT4G09030	AtAGP10	CATMA4A09010	8.18	8.88	0.70	2.80E-02
	AT1G68725	AtAGP19 (Lys-rich AGP)	CATMA1A58110	7.94	7.93	-0.01	1.00
	AT1G35230	AtAGP5	CATMA1A33365	7.34	7.49	0.15	1.00
	AT2G33790	AtAGP30	CATMA2A31980	6.95	6.57	-0.38	1.00
	AT2G23130	AtAGP17	CATMA2A21630	6.94	6.84	-0.11	1.00
	AT5G24105	AtAGP41	CATMA5A21640	6.84	6.93	0.10	1.00
fasciclin AGPs (FLAs) [x]	AT1G03870	AtFLA9	CATMA1A02720	13.28	13.25	-0.03	1.00
	AT2G45470	AtFLA8	CATMA2A43870	12.49	11.88	-0.60	7.22E-01
	AT4G12730	AtFLA2	CATMA4A12870	11.76	11.55	-0.21	1.00
	AT2G04780	AtFLA7	CATMA2A03690	10.03	9.87	-0.16	1.00
	AT3G12660	AtFLA14	CATMA3A11630	8.76	8.44	-0.33	1.00
	AT5G60490	AtFLA12	CATMA5A56200	8.62	8.36	-0.26	1.00
	AT5G44130	AtFLA13	CATMA5A39880	8.62	8.20	-0.42	1.00
	AT5G55730	AtFLA1	CATMA5A51470	8.50	8.14	-0.36	1.00
	AT5G03170	AtFLA11	CATMA5A02280	8.35	8.10	-0.25	1.00
	AT3G11700	AtFLA18	CATMA3A10640	8.14	8.08	-0.06	1.00
	AT3G60900	AtFLA10	CATMA3A53870	7.29	7.09	-0.20	1.00
	AT2G24450	AtFLA3	CATMA2A22780	7.18	7.23	0.06	1.00
	AT4G31370	AtFLA5	CATMA4A33063	7.11	6.87	-0.24	1.00
GPI-anchored peptide (GAPEP) family [y]	AT3G01950		CATMA3A00940	8.42	9.00	0.58	1.00
	AT5G50660		CATMA5A46550	7.76	7.78	0.02	1.00
	AT5G40970		CATMA5A36640	7.68	8.31	0.63	3.59E-01
	AT5G40960		CATMA5A36630	7.57	9.10	1.54	0.00E+00
	AT3G01940		CATMA3A00930	7.16	7.70	0.54	1.00
COBRA-like proteins [z]	AT5G60920	COB	CATMA5A56600	11.44	11.10	-0.34	1.00
	AT5G15630	AtCOBL4	CATMA5A13880	8.28	7.81	-0.47	1.00
	AT4G16120	AtCOBL7	CATMA4A16865	8.20	7.89	-0.31	1.00
	AT3G02210	AtCOBL1	CATMA3A01200	7.84	7.74	-0.09	1.00
	AT3G29810	AtCOBL2	CATMA3A30250	7.68	7.87	0.18	1.00
	AT4G27110	AtCOBL11	CATMA4A28710	6.95	6.73	-0.22	1.00
	AT3G16860	AtCOBL8	CATMA3A16260	6.85	6.84	-0.01	1.00

leucine-rich repeat extensins (LRXs) [aa]	AT3G24480	AtLRX4	CATMA3A24360	10.26	10.33	0.08	1.00
	AT2G15880	AtPEX3	CATMA2A14770	9.09	9.20	0.11	1.00
	AT4G33970	AtPEX4	CATMA4A35800	8.30	8.39	0.09	1.00
	AT1G62440	AtLRX2	CATMA1A51580	7.85	8.17	0.32	1.00
	AT4G18670	AtLRX5	CATMA4A19790	7.38	7.17	-0.20	1.00
	AT3G22800	AtLRX6	CATMA3A22740	6.99	7.05	0.06	1.00
	AT1G12040	AtLRX1	CATMA1A11090	6.95	6.84	-0.11	1.00
	AT1G49490	AtPEX2	CATMA1A40600	6.90	6.90	-0.01	1.00
Hyp/Pro-rich proteins (H/PRP) [ab]	AT2G21140	AtPRP2	CATMA2A19790	12.47	11.84	-0.63	3.56E-01
	AT4G38770	AtPRP4	CATMA4A40195	10.00	9.15	-0.86	7.25E-05
extensins [ac]	AT1G23720		CATMA1A22610	8.45	7.28	-1.17	1.68E-11
	AT3G54580		CATMA3A47520	8.43	7.28	-1.15	3.93E-11
	AT3G28550		CATMA3A28430	7.50	6.87	-0.63	2.88E-01
	AT1G26250		CATMA1A24480	6.94	6.68	-0.26	1.00
lignin toolbox [ad]	AT4G39330	Arath;CAD1(cinnamyl alcohol dehydrogenase)	CATMA4A40735	11.72	10.47	-1.25	0.00E+00
	AT2G30490	Arath;C4H (CYP73A5) (trans-cinnamate 4-hydroxylase)	CATMA2A28746	11.30	10.97	-0.33	1.00
	AT5G48930	Arath;HCT (hydroxycinnamoyl-CoA:shikimate/quinate hydroxycinnamoyl transferase)	CATMA5A44840	11.10	11.04	-0.05	1.00
	AT1G76790	Arath;COMT-like8 (caffeic acid O-methyltransferase)	CATMA1A66010	11.08	10.35	-0.73	9.03E-03
	AT5G54160	Arath;COMT (caffeic acid O-methyltransferase)	CATMA5A50073	10.43	9.71	-0.72	1.61E-02
	AT2G40890	Arath;C3H2 (CYP98A3) (p-coumarate 3-hydroxylase)	CATMA2A39220	10.25	9.65	-0.60	7.46E-01
	AT5G58490	Arath;CCR-like5 (cinnamoyl-CoA reductase)	CATMA5A54250	10.02	9.89	-0.13	1.00
	AT1G20480	Arath;4CL-like4 (4-coumarate CoA ligase)	CATMA1A19480	9.70	9.49	-0.21	1.00
	AT1G72680	Arath;CAD9 (cinnamyl alcohol dehydrogenase)	CATMA1A61900	8.83	8.99	0.16	1.00
	AT3G19450	Arath;CAD2 (cinnamyl alcohol dehydrogenase)	CATMA3A19045	8.74	8.23	-0.51	1.00
	AT1G15950	Arath;CCR1 (cinnamoyl-CoA reductase)	CATMA1A14998	8.66	8.53	-0.13	1.00
	AT3G10340	Arath;PAL4 (Phe ammonia lyase)	CATMA3A09340	8.43	8.51	0.08	1.00
	AT3G61990	Arath;CCoAOMT3 (caffeoyl-CoA O-methyltransferase)	CATMA3A55120	8.42	8.41	-0.01	1.00
	AT2G02400	Arath;CCR-like2 (cinnamoyl-CoA reductase)	CATMA2A01315	8.32	8.15	-0.17	1.00
	AT2G21730	Arath;CAD7 (cinnamyl alcohol dehydrogenase)	CATMA2A20370	8.06	7.67	-0.38	1.00
	AT4G34230	Arath;CAD6 (cinnamyl alcohol dehydrogenase)	CATMA4A36050	7.95	7.85	-0.09	1.00
	AT2G21890	Arath;CAD8 (cinnamyl alcohol dehydrogenase)	CATMA2A20490	7.93	7.51	-0.43	1.00
	AT1G51680	Arath;4CL1 (4-coumarate CoA ligase)	CATMA1A42785	7.92	7.97	0.05	1.00
	AT4G26220	Arath;CCoAOMT7 (caffeoyl-CoA O-methyltransferase)	CATMA4A27740	7.91	8.15	0.24	1.00
	AT3G21240	Arath;4CL2 (4-coumarate CoA ligase)	CATMA3A21086	7.79	7.67	-0.12	1.00
	AT5G04230	Arath;PAL3 (Phe ammonia lyase)	CATMA5A03420	7.76	7.55	-0.22	1.00
	AT5G63380	Arath;4CL-like8 (4-coumarate CoA ligase)	CATMA5A58930	7.75	7.31	-0.43	1.00
	AT1G20510	Arath;4CL-like1 (4-coumarate CoA ligase)	CATMA1A19510	7.72	7.51	-0.22	1.00
	AT5G04330	Arath;F5H2 (CYP84A4)	CATMA5A03540	7.61	7.53	-0.09	1.00
	AT3G21230	Arath;4CL4 (4-coumarate CoA ligase)	CATMA3A21070	7.35	7.17	-0.18	1.00
	AT2G37040	Arath;PAL1 (Phe ammonia lyase)	CATMA2A35330	7.18	7.74	0.56	1.00
	AT1G65060	Arath;4CL3 (4-coumarate CoA ligase)	CATMA1A54343	7.00	6.69	-0.32	1.00
	AT1G80820	Arath;CCR2 (cinnamoyl-CoA reductase)	CATMA1A70050	7.00	6.97	-0.04	1.00
	AT5G53810	Arath;COMT-like13 (caffeic acid O-	CATMA5A49700	7.00	7.19	0.20	1.00
	AT4G19010	Arath;4CL-like6 (4-coumarate CoA ligase)	CATMA4A20160	6.85	6.94	0.09	1.00
peroxidases [ae]	AT2G37130	AtPrx21	CATMA2A35430	14.91	13.66	-1.25	0.00
	AT4G21960	AtPrx42	CATMA4A23655	13.39	12.71	-0.68	6.22E-02
	AT3G21770	AtPrx30	CATMA3B21635	10.92	10.76	-0.16	1.00
	AT4G33870	AtPrx48	CATMA5A59590	10.83	10.23	-0.60	8.07E-01
	AT1G05260	AtPrx03	CATMA1A04113	10.78	10.30	-0.47	1.00
	AT5G64120	AtPrx71	CATMA4A35650	10.52	10.11	-0.41	1.00
	AT1G71695	AtPrx12	CATMA1A60876	10.44	9.48	-0.96	7.29E-07
	AT5G51890	AtPrx66	CATMA5A47820	9.15	8.78	-0.37	1.00
	AT5G66390	AtPrx72	CATMA5A61725	9.15	9.38	0.23	1.00
	AT4G37520	AtPrx50	CATMA4A39085	8.96	8.99	0.03	1.00
	AT5G64110	AtPrx70	CATMA5A59580	8.90	8.99	0.08	1.00
	AT2G38390	AtPrx23	CATMA2A36690	8.71	9.32	0.61	6.44E-01
	AT3G17070	AtPrx29	CATMA3A16470	8.58	8.23	-0.35	1.00
	AT5G39580	AtPrx62	CATMA5A35160	8.20	7.72	-0.48	1.00
	AT1G44970	AtPrx09	CATMA1A37915	8.07	7.83	-0.24	1.00
	AT2G41480	AtPrx25	CATMA2A39890	8.04	8.72	0.68	6.77E-02
	AT5G17820	AtPrx57	CATMA5A16105	8.01	7.07	-0.94	0.00
	AT4G37530	AtPrx51	CATMA4A39090	7.98	8.34	0.36	1.00
	AT5G40150	AtPrx63	CATMA5A35810	7.92	7.69	-0.22	1.00
	AT4G30170	AtPrx45	CATMA4A31806	7.91	7.75	-0.16	1.00
	AT2G22420	AtPrx17	CATMA2A20940	7.83	8.04	0.21	1.00
	AT5G19890	AtPrx59	CATMA5A18330	7.82	7.56	-0.26	1.00
	AT5G64100	AtPrx69	CATMA5A59575	7.42	6.80	-0.63	3.63E-01
	AT3G01190	AtPrx27	CATMA3A00185	7.41	6.73	-0.68	6.07E-02
	AT2G18980	AtPrx16	CATMA2A17560	7.35	7.19	-0.16	1.00
	AT2G24800	AtPrx18	CATMA2A23170	7.29	7.14	-0.15	1.00
	AT4G36430	AtPrx49	CATMA4A38005	7.00	7.24	0.24	1.00
	AT5G19880	AtPrx58	CATMA5A18320	6.96	6.95	-0.02	1.00
	AT5G05340	AtPrx52	CATMA5A04520	6.94	6.98	0.04	1.00
	AT5G05250	AtPrx02	CATMA1A04085	6.88	6.75	-0.14	1.00
	AT2G18150	AtPrx15	CATMA2A16820	6.81	7.17	0.36	1.00
	AT5G06730	AtPrx54	CATMA5A05910	6.79	7.14	0.35	1.00
	AT1G68850	AtPrx11	CATMA1A58250	6.72	7.06	0.34	1.00
	AT2G35380	AtPrx20	CATMA2A33510	6.63	6.95	0.32	1.00
laccases [af]	AT5G60020	AtLAC17	CATMA5A55770	10.33	9.64	-0.69	3.97E-02
	AT5G03260	AtLAC11	CATMA5A02370	10.29	10.18	-0.11	1.00
	AT2G38080	AtLAC4	CATMA2A36370	9.52	8.80	-0.72	1.40E-02
	AT2G30210	AtLAC3	CATMA2A28490	8.40	8.42	0.01	1.00
	AT2G29130	AtLAC2	CATMA2A27560	7.98	7.53	-0.45	1.00
	At3G09220	AtLAC7	CATMA3A08090	7.28	6.81	-0.47	1.00
	AT5G05390	AtLAC12	CATMA5A04570	7.18	7.24	0.06	1.00
	AT2G40370	AtLAC5	CATMA2A38650	7.04	7.13	0.10	1.00
	AT5G01190	AtLAC10	CATMA5A00220	6.89	6.80	-0.09	1.00
	AT1G18140	AtLAC1	CATMA1A17120	6.87	6.76	-0.11	1.00

**SKU-like proteins (multi-copper oxidases)** [ag]	**AT1G76160**	**SKS5**	CATMA1A65380	**11.24**	**10.98**	-0.26	1.00
	**AT1G41830**	**SKS6**	CATMA1A35650	**10.20**	**9.74**	-0.45	1.00
	**AT4G12420**	**SKU5**	CATMA4A12530	**9.97**	**9.70**	-0.27	1.00
	**AT4G22010**	**SKS4**	CATMA4A23690	**9.04**	**8.47**	-0.57	1.00
	**AT5G48450**	**SKS3**	CATMA5A44430	**9.02**	**8.66**	-0.36	1.00
	**At4G38420**	**SKS9**	CATMA4A39940	**7.48**	**7.01**	-0.47	1.00
	**AT5G51480**	**SKS2**	CATMA5A47400	**7.10**	**7.21**	0.11	1.00
**phytocyanins** [ah]	**AT4G12880**	**AtEN20 (early nodulin)**	CATMA4A13020	**11.56**	**11.94**	0.38	1.00
	**AT2G32300**	**AtUCC1 (uclacyanin)**	CATMA2A30615	**11.32**	**11.29**	-0.03	1.00
	**AT5G15350**	**AtEN22 (early nodulin)**	CATMA5A13625	**10.22**	**10.58**	0.35	1.00
	**AT1G08500**	**AtEN21 (early nodulin)**	CATMA1A07465	**9.67**	**10.04**	0.37	1.00
	**AT5G53870**	**AtEN1 (early nodulin)**	CATMA5A49760	**9.10**	**9.24**	0.14	1.00
	**AT1G72230**	**AtUCC8 (uclacyanin)**	CATMA1A61460	**8.91**	**9.34**	0.43	1.00
	**AT3G20570**	**AtEN14 (early nodulin)**	CATMA3A20300	**8.74**	**8.83**	0.09	1.00
	**AT4G28360**	**AtEN2 (early nodulin)**	CATMA4A30000	**8.57**	**8.65**	0.09	1.00
	**AT4G31840**	**AtEN13 (early nodulin)**	CATMA4A33490	**8.51**	**8.82**	0.31	1.00
	**AT3G60280**	**AtUCC3 (uclacyanin)**	CATMA3A53305	**8.29**	**8.40**	0.11	1.00
	**AT4G27520**	**AtEN12 (early nodulin)**	CATMA4A29090	**8.02**	**8.46**	0.44	1.00
	**AT2G27035**	**AtEN17 (early nodulin)**	CATMA2A25400	**7.83**	**8.07**	0.23	1.00
	**AT3G27200**	**AtUCC6 (uclacyanin)**	CATMA3A27010	**7.77**	**8.01**	0.24	1.00
	**AT2G25060**	**AtEN7 (early nodulin)**	CATMA2A23390	**7.74**	**7.75**	0.02	1.00
	**AT4G33930**	**AtEN26 (early nodulin)**	CATMA4A35740	**7.47**	**7.30**	-0.17	1.00
	**AT5G25090**	**AtEN6 (early nodulin)**	CATMA5A22800	**7.43**	**7.63**	0.20	1.00
	**AT5G20230**	**AtSTC1 (stellacyanin)**	CATMA5A18670	**7.32**	**8.73**	1.41	0.00E+00
	**AT5G26330**	**AtSTC3 (stellacyanin)**	CATMA5A24030	**7.26**	**7.30**	0.04	1.00
	**AT2G02850**	**AtPNC (plantacyanin)**	CATMA2A01770	**7.26**	**7.22**	-0.04	1.00
	**AT5G57920**	**AtEN3 (early nodulin)**	CATMA5A53680	**7.05**	**7.10**	0.06	1.00
	**AT5G07475**	**AtUCC5 (uclacyanin)**	CATMA5A06680	**6.89**	**6.94**	0.05	1.00
**subtilases** [ai]	**AT3G14067**	**AtSBT1.4**	CATMA3A13290	**12.34**	**11.18**	-1.16	2.25E-11
	**AT2G05920**	**AtSBT1.8**	CATMA2A04690	**12.30**	**11.50**	-0.81	6.10E-04
	**AT5G67360**	**AtSBT1.7 (ARA12)**	CATMA5A62810	**12.07**	**11.68**	-0.39	1.00
	**AT5G59090**	**AtSBT4.12**	CATMA5A54840	**11.25**	**10.12**	-1.13	1.46E-10
	**AT5G51750**	**AtSBT1.3**	CATMA5A47680	**11.18**	**9.81**	-1.37	0.00E+00
	**AT3G14240**	**AtSBT1.5**	CATMA3A13520	**10.36**	**10.16**	-0.20	1.00
	**AT1G20160**	**AtSBT5.2**	CATMA1A19170	**9.60**	**9.43**	-0.17	1.00
	**AT4G00230**	**AtSBT4.14 (XSP11)**	CATMA4A00245	**9.06**	**9.08**	0.01	1.00
	**AT4G34980**	**AtSBT1.6**	CATMA4A36760	**8.80**	**8.63**	-0.17	1.00
	**AT2G04160**	**AtSBT5.3 (AIR3)**	CATMA2A03130	**8.53**	**7.61**	-0.92	3.86E-06
	**AT5G19660**	**AtSBT6.1**	CATMA5A18090	**7.78**	**7.77**	0.00	1.00
	**AT1G30600**	**AtSBT2.1**	CATMA1A28650	**7.71**	**7.56**	-0.15	1.00
	**AT5G44530**	**AtSBT2.3**	CATMA5A40320	**7.37**	**7.15**	-0.22	1.00
	**AT4G21326**	**AtSBT3.12**	CATMA4A22930	**7.22**	**7.24**	0.02	1.00
	**AT2G19170**	**AtSBT2.5**	CATMA2A17780	**7.15**	**6.90**	-0.26	1.00
	**AT4G20850**	**AtSBT6.2**	CATMA4A22360	**7.14**	**7.21**	0.07	1.00
	**AT5G45650**	**AtSBT5.6**	CATMA5A41630	**7.12**	6.77	-0.34	1.00
**pectin methylesterase/invertase inhibitors** [aj] **(PMEIs)**	**AT3G12880**		CATMA3A11840	**9.41**	**9.33**	-0.08	1.00
	**AT4G25260**		CATMA4A26950	**9.37**	**9.86**	0.49	1.00
	**AT5G64620**		CATMA5A60045	**9.00**	**9.07**	0.07	1.00
	**AT2G31430**		CATMA2A29670	**8.94**	**8.61**	-0.33	1.00
	**AT5G62350**		CATMA5A57950	**8.74**	**8.97**	0.23	1.00
	**AT3G47670**		CATMA3A40660	**8.48**	**8.40**	-0.08	1.00
	**AT3G47380**		CATMA3A40380	**8.21**	**8.39**	0.18	1.00
	**AT4G12390**		CATMA4A12500	**8.18**	**8.13**	-0.05	1.00
	**AT1G47960**		CATMA1A39055	**7.79**	**8.43**	0.63	2.97E-01
	**AT3G49330**		CATMA3A42360	**7.76**	**7.93**	0.18	1.00
	**AT2G01610**		CATMA2A00660	**7.75**	**8.02**	0.27	1.00
	**AT1G23205**		CATMA1A22290	**7.54**	**8.00**	0.46	1.00
	**AT1G14890**		CATMA1A13920	**7.50**	**7.79**	0.30	1.00
	**AT3G17220**		CATMA3A16630	**7.32**	**7.61**	0.28	1.00
	**AT3G62820**		CATMA3A55990	**7.25**	**7.31**	0.07	1.00
	**AT1G70720**		CATMA1A60000	**7.07**	**7.23**	0.16	1.00
	**AT5G20740**		CATMA5A19270	**7.05**	**7.04**	-0.01	1.00
	**AT1G62760**		CATMA1A51880	**6.97**	**7.16**	0.19	1.00

a: http://cellwall.genomics.purdue.edu/families/4-6-5.html
b: http://cellwall.genomics.purdue.edu/families/1-1.html
c: http://cellwall.genomics.purdue.edu/families/2-3-1.html
d: http://cellwall.genomics.purdue.edu/families/2-3-5.html
e: http://cellwall.genomics.purdue.edu/families/2-3-4.html
f: http://cellwall.genomics.purdue.edu/families/1-1.html
g: http://cellwall.genomics.purdue.edu/families/2-3-2.html
h: http://www.cazy.org/fam/GT77.html, Egelhund *et al.* (2004)
i: http://cellwall.genomics.purdue.edu/families/2-2.html
j: http://cellwall.genomics.purdue.edu/families/2-4.html
k: http://cellwall.genomics.purdue.edu/families/3-1.html
l: http://cellwall.genomics.purdue.edu/families/4-3-2-1.html
m: http://labs.plantbio.cornell.edu/xth/genes.htm
n: http://cellwall.genomics.purdue.edu/families/4-3-2-2.html
o: http://cellwall.genomics.purdue.edu/families/4-1-2.html
p: http://cellwall.genomics.purdue.edu/families/4-3-3.html
q: http://cellwall.genomics.purdue.edu/families/4-3-1-1.html
r: http://cellwall.genomics.purdue.edu/families/4-5-1.html
s: http://cellwall.genomics.purdue.edu/families/4-5-2.html
t: http://cellwall.genomics.purdue.edu/families/4-4-1.html
u: http://cellwall.genomics.purdue.edu/families/4-4-2.html
v: http://www.bio.psu.edu/expansins/index.htm
w: Schultz *et al.* (2002), Johnson *et al.* (2003), van Engel and Roberts (2003), Liu and Mehdy (2007)
x: Schultz *et al.* (2002)
y: http://cellwall.genomics.purdue.edu/families/6-4-10.html
z: Roudier *et al.* (2005)
aa: Baumberger *et al.* (2003)
ab: Fowler *et al.* (1999)
ac: our own annotation
ad: Raes *et al.* (2003)
ae: http://peroxibase.isb-sib.ch/index.php
af: Pourcel *et al.* (2005), McCaig *et al.* (2005)
ag: Jacobs and Roe (2005)
ah: Nersissian and Ship (2002)
ai: http://csbdb.mpimp-golm.mpg.de/csbdb/dbcawp/psdb/pub/sgenes.html
aj: Dr Jérôme Pelloux (Amiens University, France), personal communication
