# Supplementary material for: Cell wall biogenesis of Arabidopsis thaliana elongating cells: transcriptomics complements proteomics
Source: BMC Genomics. 2009 Oct 31;10:505. doi: 10.1186/1471-2164-10-505 (PMC2774874; doi:10.1186/1471-2164-10-505)
Supplement: Additional file 2 — Cell Wall Genes (CWGs) with high levels of transcripts in either 5- or 11-day-old hypocotyls. The data provided represent the CWGs having high levels of transcripts. [file 1471-2164-10-505-S2.pdf]

Additional file 2. Cell Wall Genes (CWGs) with high levels of transcripts in either 5- or 11-day-old hypocotyls.

CWGs were selected according to several papers and databases as mentioned in Methods. The intensity of the signal is expressed as log_2. The log_2 of ratios between the levels of transcripts at 11-days and 5-days as well as the Bonferroni p-values are indicated. CWGs are classified family wise.

Functional classes	AGI number	Predicted or known gene function	5-days	11-days	log_2 of ratio 11-days / 5-days	p-value
**Substrate generation**						
nucleotide-sugar interconversion pathway	AT5G39320	UGD1	11.58	10.81	-0.77	2.19E-03
	AT1G08200	AXS2	10.71	10.61	-0.10	1.00
	AT5G15490	UGD3	10.50	10.00	-0.51	1.00
**Lignin toolbox**						
	AT4G39330	CAD1(cinnamyl alcohol dehydrogenase)	11.72	10.47	-1.25	0.00E+00
	AT2G30490	C4H (CYP73A5) (trans-cinnamate 4-hydroxylase)	11.30	10.97	-0.33	1.00
	AT5G48930	HCT (hydroxycinnamoyl-CoA:shikimate/quinate hydroxycinnamoyl transferase)	11.10	11.04	-0.05	1.00
	AT1G76790	COMT-like8 (caffeic acid O-methyltransferase)	11.08	10.35	-0.73	9.03E-03
	AT5G54160	COMT (caffeic acid O-methyltransferase)	10.43	9.71	-0.72	1.61E-02
	AT2G40890	C3H2 (CYP98A3) (p-coumarate 3-hydroxylase)	10.25	9.65	-0.60	7.46E-01
	AT5G58490	CCR-like5 (cinnamoyl-CoA reductase)	10.02	9.89	-0.13	1.00
	AT5G18900	prolyl-4-hydroxylase	10.24	10.29	0.05	1.00
**Synthesis of polysaccharides**						
glycosyl transferases (GTs)	AT5G09870	GT2 (AtCESA5)	10.43	9.65	-0.78	1.64E-03
	AT5G05170	GT2 (AtCESA3)	10.40	9.88	-0.52	1.00
	AT4G39350	GT2 (AtCESA2)	10.23	9.52	-0.72	1.78E-02
	AT4G32410	GT2 (AtCESA1)	10.05	9.53	-0.52	1.00
	AT1G77130	GT8 (Group A)	10.11	9.79	-0.32	1.00
	AT4G00300	GT31 (Group A)	10.06	9.55	-0.51	1.00
	AT2G22900	GT34	10.26	9.71	-0.55	1.00
	AT2G28110	GT47 (FRA8)	11.69	11.26	-0.43	1.00

	AT1G19360	GT77	10.37	10.07	-0.29	1.00
**Vesicle trafficking**						
	AT1G69460	emp24/gp25L/p24 family protein	10.88	10.55	0.33	1.00
**Modification of polysaccharides**						
glycosyl hydrolases (GHs)	AT1G64390	GH9 (endoglucanases)	10.68	10.19	-0.49	1.00
	AT4G02290	GH9 (endoglucanases)	11.10	10.08	-1.02	1.57E-07
	AT4G14130	GH16 (AtXTH15)	13.61	12.31	-1.31	0.00E+00
	AT2G06850	GH16 (AtXTH4)	13.27	12.62	-0.65	1.96E-01
	AT4G30290	GH16 (AtXTH19)	12.37	12.15	-0.22	1.00
	AT1G32170	GH16 (AtXTH30)	11.24	11.17	-0.06	1.00
	AT1G11545	GH16 (At-XTH8)	10.48	10.34	-0.14	1.00
	AT2G01850	GH16 (At-XTH27)	10.42	10.34	-0.08	1.00
	AT4G30270	GH16 (AtXTH24)	9.88	10.14	0.25	1.00
	AT5G42100	GH17 (Group C)	10.36	10.13	-0.22	1.00
	AT3G04010	GH17 (Group B)	10.25	9.24	-1.01	6.09E-08
	AT5G58090	GH17 (Group B)	9.83	10.16	0.33	1.00
	AT4G23820	GH28 (Group A)	10.23	9.89	-0.34	1.00
	AT5G56870	GH35 (beta galactosidases) (AtBGAL4)	13.37	12.75	-0.62	4.79E-01
	AT1G45130	GH35 (beta galactosidases) (AtBGAL5)	11.77	11.16	-0.61	5.91E-01
carbohydrate esterases (CEs)	AT2G43050	CE8 (pectin methylesterases)	12.72	11.24	-1.48	0.00E+00
	AT3G49220	CE8 (pectin methylesterases)	11.84	11.15	-0.69	3.94E-02
	AT3G14310	CE8 (pectin methylesterases) AtPME3	11.66	10.82	-0.84	1.44E-04
	AT3G05910	CE13 (pectin acylesterases)	10.55	10.41	-0.15	1.00
carbohydrate lyases (CLs)	AT5G48900	PL1 (pectate lyases) (Group A)	11.58	10.93	-0.65	1.52E-01
	AT3G07010	PL1 (pectate lyases) (Group A)	11.30	10.91	-0.39	1.00E+00
	AT1G04680	PL1 (pectate lyases) (Group A)	11.30	10.97	-0.33	1.00
	AT1G67750	PL1 (pectate lyases) (Group A)	10.86	10.36	0.67	1.00
	AT4G24780	PL1 (pectate lyases) (Group A)	9.69	10.36	0.67	9.00E-02
expansins	AT5G02260	AtEXPA9 (alpha expansin)	12.95	12.54	-0.41	1.00
	AT1G69530	AtEXPA1 (alpha expansin)	11.10	11.63	0.52	1.00
	AT2G40610	AtEXPA8 (alpha expansin)	10.49	10.40	-0.09	1.00
	AT4G28250	AtEXPB3 (beta expansin)	10.01	10.66	0.65	1.97E-01

**Structural proteins**						
Hyp/Pro rich proteins (H/PRP)	AT2G21140	AtPRP2	12.47	11.84	-0.63	3.56E-01
	AT4G38770	AtPRP4	10.00	9.15	-0.86	7.25E-05
LRR-extensins	AT3G24480	AtLRX4	10.26	10.33	0.08	1.00
**Oxido-reductases**						
peroxidases	AT2G37130	AtPrx21	14.91	13.66	-1.25	0.00
	AT4G21960	AtPrx42	13.39	12.71	-0.68	6.22E-02
	AT3G21770	AtPrx30	10.92	10.76	-0.16	1.00
	AT4G33870	AtPrx48	10.38	10.23	-0.60	8.07E-01
	AT1G05260	AtPrx03	10.78	10.30	-0.47	1.00
	AT5G64120	AtPrx71	10.52	10.11	-0.41	1.00
	AT1G71695	AtPrx12	10.44	9.48	-0.96	7.29E-07
phytocyanins	AT4G12880	AtEN20 (early nodulin)	11.56	11.94	0.38	1.00
	AT2G32300	AtUCC1 (uclacyanin)	11.32	11.29	-0.03	1.00
	AT5G15350	AtEN22 (early nodulin)	10.22	10.58	0.35	1.00
	AT1G08500	AtEN21 (early nodulin)	9.67	10.04	0.37	1.00
SKU-like proteins (multi-copper oxidases)	AT1G76160	SKS5	11.24	10.98	-0.26	1.00
	AT1G41830	SKS6	10.20	9.74	-0.45	1.00
**Proteins possibly involved in signaling**						
arabinogalactan proteins (AGPs)	AT2G14890	AtAGP9	12.43	12.55	0.12	1.00
	AT5G11740	AtAGP15 (AG peptide)	12.04	12.70	0.66	1.26E-01
	AT1G28290	AtAGP31	11.98	11.89	-0.09	1.00
	AT3G13520	AtAGP12 (AG peptide)	11.93	12.97	1.04	1.23E-08
	AT5G10430	AtAGP4	11.76	11.86	0.10	1.00
	AT5G53250	AtAGP22 (AG peptide)	9.98	10.94	0.96	0.00
	AT3G61640	AtAGP20 (AG peptide)	9.91	10.65	0.74	6.96E-03
fasciclin AGPs (FLAs)	AT1G03870	AtFLA9	13.28	13.25	-0.03	1.00
	AT2G45470	AtFLA8	12.49	11.88	-0.60	7.22E-01
	AT4G12730	AtFLA2	11.76	11.55	-0.21	1.00
	AT2G04780	AtFLA7	10.03	9.87	-0.16	1.00
**Proteases**						
subtilases	AT3G14067	AtSBT1.4	12.34	11.18	-1.16	2.25E-11
	AT2G05920	AtSBT1.8	12.30	11.50	-0.81	6.10E-04
	AT5G67360	AtSBT1.7 (ARA12)	12.07	10.12	-0.39	1.00
	AT5G59090	AtSBT4.12	11.25	10.12	-1.13	1.46E-10
	AT5G51750	AtSBT1.3	11.18	9.81	-1.37	0.00E+00
	AT3G14240	AtSBT1.5	10.36	10.16	-0.20	1.00
**Miscellaneous**						
COBRA-like	AT5G60920	COB	11.44	11.10	-0.34	1.00
