# Supplementary material for: Cell wall biogenesis of Arabidopsis thaliana elongating cells: transcriptomics complements proteomics
Source: BMC Genomics. 2009 Oct 31;10:505. doi: 10.1186/1471-2164-10-505 (PMC2774874; doi:10.1186/1471-2164-10-505)
Supplement: Additional file 3 — Secretory Pathway Genes (SPGs) with moderate or high levels of transcripts in 5- and 11-day-old etiolated hypocotyls. The data provided represent the levels of transcripts of SPGs and the corresponding statistical analyses. [file 1471-2164-10-505-S3.pdf]

Additional file 3. Secretory Pathway Genes (SPGs) with moderate or high levels of transcripts in 5- and 11-day-old hypocotyls. SPGs were annotated using bioinformatic softwares for sub-cellular localization and functional domains as described in Methods. The intensity of the signal is expressed as log_2, the ratio between the levels of transcripts at 11-days and 5-days as well as the p-value are indicated. CWGs are classified by gene family and level of transcript: low level in blue (between background and 9), moderate level in green (between 9 and 10) and high level in red (higher than 10). Significant Bonferroni p-values are highlighted in green (p<5%).

Genes encoding proteins predicted to be outside the cell are highlighted in yellow, proteins predicted to secreted and to have trans-membrane domains are in pink, and proteins predicted to be secreted and to have a GPI anchor are in dark yellow. Genes encoding proteins identified in the proteomic study (Irshad *et al.*, submitted for publication) performed with the same plant material are in bold characters.

	Functional class	Gene family	AGI number	gene name	CATMA probe	5-days	11-days	log_2 of ratio 11-days / 5-days	p-value
1	**Proteins acting on carbohydrates**	glycoside hydrolase family 1 (beta-glucosidase)	AT3G60130		CATMA3A53140	11.42	10.59	-0.83	2.39E-04
2			AT3G18080		CATMA3A17550	10.95	10.51	-0.44	1.00
3			AT5G26000		CATMA5A23655	10.15	9.39	-0.76	4.17E-03
4			AT1G02850		CATMA1A01780	9.34	10.00	0.65	1.50E-01
5			AT3G21360		CATMA3A21250	9.30	10.06	0.76	3.60E-03
6			AT2G44450		CATMA2A42880	9.27	9.09	-0.18	1.00
7			AT3G60140	DIN2 (DARK INDUCIBLE 2)	CATMA3A53150	8.01	10.03	2.01	0.00E+00
8			AT1G52400		CATMA1A43455	7.77	9.15	1.37	0.00E+00
9		glycoside hydrolase family 3	AT5G49360		CATMA5A45350	13.31	13.07	-0.24	1.00
10			AT5G10560		CATMA5A09290	10.86	10.36	-0.49	1.00
11			AT5G20950		CATMA5A19500	10.10	9.93	-0.17	1.00
12		glycoside hydrolase family 9	AT1G64390		CATMA1A53700	11.38	10.66	-0.72	1.41E-02
13			AT4G02290		CATMA4A02570	10.42	9.44	-0.97	4.35E-07
14			AT5G49720	AtKOR	CATMA5A45658	9.81	9.62	-0.19	1.00
15			AT1G70710	AtCEL1	CATMA1A59990	9.90	9.29	-0.62	5.15E-01
16		glycoside hydrolase family 16 (xyloglucan endotransglycosidases/hydrolases)	AT4G14130	AtXTH15	CATMA4A14375	13.61	12.31	-1.31	0.00E+00
17			**AT2G06850**	**AtXTH4**	CATMA2A05540	13.27	12.62	-0.65	1.96E-01
18			AT4G30290	AtXTH19	CATMA4A31900	12.37	12.15	-0.22	1.00
19			AT1G32170	AtXTH30	CATMA1A30510	11.24	11.17	-0.06	1.00
20			AT1G11545	AtXTH8	CATMA1A10510	10.48	10.34	-0.14	1.00
21			AT2G01850	AtXTH27	CATMA2A00905	10.42	10.34	-0.08	1.00
22			**AT3G44990**	**AtXTH31**	CATMA3A38015	10.10	8.76	-1.35	0.00E+00
23			AT4G30270	AtXTH24	CATMA4A31885	9.88	10.14	0.25	1.00
24			AT1G14720	AtXTH28	CATMA1A13765	9.72	9.73	0.00	1.00
25			**AT5G13870**	**AtXTH5**	CATMA5A12105	9.02	8.63	-0.39	1.00
26		glycoside hydrolase family 17	AT5G42100		CATMA5A37820	10.36	10.13	-0.22	1.00
27			AT3G04010		CATMA3A02980	10.25	9.24	-1.01	6.09E-08
28			AT5G58090		CATMA5A53850	9.83	10.16	0.33	1.00
29			AT2G05790		CATMA2A04550	9.71	9.45	-0.26	1.00
30			AT5G42720		CATMA5A38510	9.45	9.08	-0.37	1.00
31			AT3G07320		CATMA3A06560	9.33	8.76	-0.57	1.00
32			AT5G20330		CATMA5A18800	9.07	9.09	0.02	1.00
33		glycoside hydrolase family 19 (chitinase class I)	AT1G05850	ELP: ECTOPIC DEPOSITION OF LIGNIN IN PITH	CATMA1A04870	13.22	12.68	-0.53	1.00
34			AT2G43590	glycoside hydrolase family 19	CATMA2B42010	9.61	8.31	-1.30	0.00E+00
35		glycoside hydrolase family 20	**AT3G55260**		CATMA3A46320	10.40	9.90	-0.51	1.00
36		glycoside hydrolase family 27	AT5G08380		CATMA5A07660	9.14	9.11	-0.03	1.00
37		glycoside hydrolase family 28	AT4G23820		CATMA4A25640	10.23	9.89	-0.34	1.00
38			AT3G57790		CATMA3A50830	9.12	8.79	-0.33	1.00
39		glycoside hydrolase family 31	**AT1G68560**	**XYL1 (alpha-xylosidase)**	CATMA1A57930	10.97	10.57	-0.41	1.00
40			AT5G63840	RSW3 (RADIAL SWELLING 3)	CATMA5A59360	9.57	9.58	0.00	1.00
41		glycoside hydrolase family 35 (beta-galactosidase)	AT5G56870	AtBGAL4	CATMA5A52625	13.37	12.75	-0.62	4.79E-01
42			AT1G45130	AtBGAL5	CATMA1A37985	11.77	11.16	-0.61	5.91E-01
43		glycoside hydrolase family 38 (alpha-mannosidase)	AT3G26720		CATMA3A26465	11.89	11.22	-0.67	8.25E-02
44			AT5G13980		CATMA5A12210	10.16	9.63	-0.52	1.00
45		glycoside hydrolase family 47	AT1G27520		CATMA1A25750	9.93	10.02	0.09	1.00
46		pectate lyase family 1	AT5G48900		CATMA5A44810	11.58	10.93	-0.65	1.52E-01
47			AT3G07010		CATMA3A06210	11.30	10.91	-0.39	1.00
48			AT1G04680		CATMA1A03520	11.30	10.97	-0.33	1.00
49			AT1G67750		CATMA1A57110	10.89	10.35	-0.54	1.00
50			AT4G24780		CATMA4A26470	9.69	10.36	0.67	9.00E-02
51		carbohydrate esterase family 8 (pectin methylesterases)	AT2G43050		CATMA2A41450	12.72	11.24	-1.48	0.00E+00
52			AT3G49220		CATMA3A42250	11.84	11.15	-0.69	3.94E-02
53			**AT3G14310**	**AtPME3**	CATMA3A13580	11.66	10.82	-0.84	1.44E-04
54			AT1G53840		CATMA1A44933	9.72	9.75	0.03	1.00
55		carbohydrate esterase family 13 (pectin acylesterases)	AT3G05910		CATMA3A04940	10.55	10.41	-0.15	1.00
56		carbohydrate esterase family 19 (pectin acylesterases)	AT4G19410		CATMA4A20590	9.23	8.97	-0.26	1.00
57			AT5G57655	homologous to xylose isomerase	CATMA5A53360	11.09	11.09	0.00	1.00
58		glycosyl transferase family 2 (cellulose synthases)	AT5G09870	AtCESA5	CATMA5A08637	10.43	9.65	-0.78	1.64E-03
59			AT5G05170	AtCESA3	CATMA5A04375	10.40	9.88	-0.52	1.00
60			AT4G39350	AtCESA2	CATMA4A40743	10.23	9.52	-0.72	1.78E-02
61			AT4G32410	AtCESA1	CATMA4A34150	10.05	9.53	-0.52	1.00
62		glycosyl transferase family 48 (callose synthases)	**AT1G05570**	**AtCALS1**	CATMA1A04500	9.77	9.06	-0.72	1.84E-02
63			AT2G31960	AtGSL3	CATMA2A30240	9.51	9.25	-0.26	1.00
64		expansins	**AT5G02260**	**AtEXPA9**	CATMA5A01340	12.95	12.54	-0.41	1.00
65			AT1G69530	AtEXPA1	CATMA1A58850	11.10	11.63	0.52	1.00
66			AT2G40610	AtEXPA8	CATMA2A38930	10.49	10.40	-0.09	1.00
67			AT2G28950	AtEXPA6	CATMA2B27340	9.73	9.81	0.08	1.00
68			**AT1G20190**	**AtEXPA11**	CATMA1A19190	9.56	9.31	-0.24	1.00
69			AT4G28250	AtEXPB3	CATMA4A29890	10.01	10.66	0.65	1.97E-01

	Functional class	Gene family	AGI number	gene name	CATMA probe	5-days	11-days	log_2 of ratio 11-days / 5-days	p-value
1	**Proteases**	cysteine proteases (papain family)	AT5G60360		CATMA5A56105	12.85	12.44	-0.41	1.00
2			**AT4G01610**		CATMA4A01830	12.70	12.50	-0.20	1.00
3			**AT5G43060**		CATMA5A38880	12.17	11.53	-0.65	0.19
4			AT1G62290		CATMA1A51380	11.63	11.23	-0.41	1.00
5			AT4G16190		CATMA4A16980	9.96	9.94	-0.02	1.00
6			AT1G20850	XCP2 (XYLEM CYSTEINE PEPTIDASE 2)	CATMA1A19916	9.64	9.12	-0.52	1.00
7			**AT4G36880**		CATMA4A38470	9.40	8.26	-1.14	6.74E-11
8			AT3G43960		CATMA3A36840	9.18	8.40	-0.78	1.71E-03
9		serine proteases (proteins homologous to serine proteases)	AT3G14067	AtSBT1.4	CATMA3A13290	12.34	11.18	-1.16	2.25E-11
10			AT2G05920	AtSBT1.8	CATMA2A04690	12.30	11.50	-0.81	6.10E-04
11			AT5G67360	AtSBT1.7, ARA12	CATMA5A62810	12.07	11.68	-0.39	1.00
12			AT5G59090	AtSBT4.12	CATMA5A54840	11.25	10.12	-1.13	1.46E-10
13			AT5G51750	AtSBT1.3	CATMA5A47680	11.18	9.81	-1.37	0.00
14			AT3G14240	AtSBT1.5	CATMA3A13520	10.36	10.16	-0.20	1.00
15			AT1G20160	AtSBT5.2	CATMA1A19170	9.60	9.43	-0.17	1.00
16			At2G00230	AtSBT4.14	CATMA4A00245	9.06	9.08	0.01	1.00
17			AT4G36190	serine carboxypeptidase S28	CATMA4A37850	9.81	9.49	-0.32	1.00

18		aspartyl proteases	AT1G11910		CATMA1A10930	13.37	13.00	-0.37	1.00
19			AT1G66180		CATMA1A55440	11.80	11.06	-0.74	7.96E-03
20			**AT5G10770**		CATMA5A09510	11.73	9.55	-2.17	0.00E+00
21			AT3G54400		CATMA3A47330	10.30	9.85	-0.45	1.00
22			AT5G19120		CATMA5A17530	10.12	10.03	-0.09	1.00
23			AT3G61820		CATMA5A57420	10.10	10.19	0.09	1.00
24			AT3G52500		CATMA3A45410	9.46	8.75	-0.72	1.79E-02
25			AT3G25700		CATMA3A25480	9.17	8.66	-0.51	1.00
26			AT1G08210		CATMA1A07200	9.11	9.39	0.29	1.00
27			AT4G16563		CATMA4A17500	9.02	8.64	-0.38	1.00
28		peptidases	**AT4G32940**	**asparaginyl peptidase**	CATMA4A34690	12.75	11.82	-0.93	3.04E-06
29			AT4G38220	metallopeptidases M20/M25/M40	CATMA4A39730	10.76	9.95	-0.80	7.27E-04
30			AT1G78680	carboxypeptidase	CATMA1A67745	10.58	10.09	-0.49	1.00
31			AT3G10450	SCPL7 (serine carboxypeptidase)	CATMA3A09450	10.43	10.26	-0.17	1.00
32			AT5G56660	metallopeptidases M20/M25/M40	CATMA5A52456	10.27	10.17	-0.11	1.00
33			AT5G08260	SCPL35 (serine carboxypeptidase)	CATMA5A07520	10.10	9.46	-0.64	2.66E-01
34			AT5G19740	M28 family	CATMA5A18160	9.75	9.55	-0.20	1.00
35			AT3G01720	metallopeptidase	CATMA3A00730	9.73	9.10	-0.63	3.21E-01
36			AT3G02875	M20 family	CATMA3A01800	9.12	9.09	-0.03	1.00
37			AT4G01320	M48 family (AtSTE24)	CATMA4A01520	9.02	8.86	-0.15	1.00
1	**Proteins possibly involved in signaling**	arabinogalactan proteins (AGPs)	AT2G14890	AtAGP9	CATMA3A12660	12.43	12.55	0.12	1.00
2			AT5G11740	AtAGP15	CATMA5A10540	12.04	12.70	0.66	1.26E-01
3			AT3G13520	AtAGP12	CATMA3A12660	11.93	12.97	1.04	1.23E-08
4			AT5G10430	AtAGP4	CATMA5A09183	11.76	11.86	0.10	1.00
5			AT5G40730	AtAGP24	CATMA5A36430	11.06	11.55	0.49	1.00
6			AT5G53250	AtAGP22	CATMA5A49170	9.98	10.94	0.96	6.83E-07
7			AT3G61640	AtAGP20	CATMA3A54770	9.91	10.65	0.74	6.96E-03
8			AT5G64310	AtAGP1	CATMA5A59763	9.62	9.86	0.24	1.00
9			AT2G47930	AtAGP26	CATMA2A46360	9.07	9.62	0.55	1.00
10		fasciclin-AGPs (FLAs)	AT1G03870	AtFLA9	CATMA1A02720	13.28	13.25	-0.03	1.00
11			AT2G45470	AtFLA8	CATMA2A22160	12.49	11.88	-0.60	7.22E-01
12			AT4G12730	AtFLA2	CATMA4A12870	11.76	11.55	-0.21	1.00
13			AT2G04780	AtFLA7	CATMA2A03690	10.03	9.87	-0.16	1.00
14			AT3G52370	AtFLA15	CATMA3A45310	9.18	8.39	-0.79	1.22E-03
15		GPI-anchored peptides (GAPEPs)	AT3G01950		CATMA3A00940	8.42	9.00	0.58	1.00
16		hormones	AT3G49780	AtPSK3 (PHYTOSULFOKINE 3 PRECURSOR)	CATMA3A42820	9.00	10.13	1.13	1.01E-10
17		expressed proteins	AT2G23810	tetraspannin domain, signal transduction?	CATMA2A22170	11.42	11.39	-0.02	1.00
18			AT1G32400	tetraspannin domain, signal transduction?	CATMA1A30780	11.22	11.17	-0.05	1.00
19		proteins homologous to receptor-kinases (RLKs)	AT2G01820	LRR IX subfamily	CATMA2A00870	11.76	11.28	-0.48	1.00
20			AT1G09970	LRR-XI subfamily	CATMA1A08840	11.42	11.34	-0.08	1.00
21			AT1G53730	AtSRF6 (RLK/Pelle, LRR-V subfamily/STRUBBELIG family)	CATMA1A44820	10.84	10.34	-0.50	1.00
22			AT1G27190	RLK/Pelle, LRR-Xa subfamily	CATMA1A25440	10.73	10.70	-0.04	1.00
23			AT2G37710	AtLRK1/AtLecRK-e (RLK/Pelle, L-Lectin subfamily)	CATMA2A35995	10.42	10.22	-0.20	1.00
24			AT1G28440	HSL1 (HAESA-LIKE 1) (LRR XI subfamily)	CATMA1A26640	10.20	9.56	-0.65	1.74E-01
25			AT1G69270	AtRPK1	CATMA1A58565	10.11	9.51	-0.60	8.64E-01
26			AT1G31420	LRR XIII subfamily	CATMA1A29670	10.09	9.65	-0.45	1.00
27			AT3G49670	BAM1 (BIG APICAL MERISTEM 1) (LRR VII subfamily)	CATMA3A42710	10.09	9.39	-0.70	3.09E-02
28			AT1G51940	LysM subfamily	CATMA1A43060	9.90	9.58	-0.33	1.00
29			AT2G26330	ERECTA (LRR XIII subfamily)	CATMA2A24675	9.90	8.99	-0.90	9.91E-06
30			AT1G48480	RLK1 (RECEPTOR-LIKE KINASE 1) (LRRIII subfamily)	CATMA1A39580	9.60	9.74	-0.13	1.00
31			AT1G08590	CLAVATA1 (LRR XI subfamily)	CATMA1A07510	9.60	8.76	-0.85	1.12E-04
32			AT4G27300	SD-1 (S-locus glycoprotein-like domain)	CATMA4A28880	9.54	9.49	-0.05	1.00
33			AT5G16000	RLK/Pelle, LRR-II subfamily	CATMA5A14280	9.52	9.37	-0.15	1.00
34			AT2G02220	PSKR1 (PHYTOSULFOKIN RECEPTOR 1) (LRR X subfamily)	CATMA2A01270	9.40	9.06	-0.34	1.00
35			AT2G39360	RLK/Pelle, CrRLK1L-1 subfamily	CATMA2A37610	9.39	8.93	-0.46	1.00
36			AT5G16590	LRR III subfamily	CATMA5A14910	9.38	8.88	-0.50	1.00
37			AT3G23150	ETR2 (ETHYLENE RESPONSE 2)	CATMA3A23140	9.35	8.72	-0.63	3.14E-01
38			AT1G07650	LRR subfamily	CATMA1A06710	9.34	9.80	-0.46	1.00
39			AT2G39180	AtCRR2	CATMA2A37410	9.27	8.78	-0.50	1.00
40			AT1G70530	DUF26 subfamily	CATMA1A59790	9.23	9.17	-0.06	1.00
41			AT1G73980	PEPR1 (PEP1 RECEPTOR 1) (LRR XI subfamily)	CATMA1A62310	9.11	8.83	-0.28	1.00
42			AT5G48380	LRR X subfamily	CATMA5A44340	9.04	8.86	-0.18	1.00
43			AT1G51890	LRR I subfamily	CATMA1B43000	8.89	9.10	0.22	1.00
44			AT3G24660	TMKL1 (TRANSMEMBRANE KINASE-LIKE 1) (LRR III receptor-like kinase)	CATMA3A24616	8.79	9.05	0.27	1.00
1	**Structural proteins**	glycine-rich proteins (GRPs)	AT2G05380	AtGRP3	CATMA2A04205	12.94	12.96	0.02	1.00
2			AT2G05540		CATMA2A04310	12.45	13.37	0.92	3.93E-06
3			AT2G05520		CATMA2A04295	12.32	12.60	0.28	1.00
4			AT4G01985		CATMA4B02280	9.03	9.08	0.06	1.00
5			AT5G46730		CATMA5A42740	8.75	9.22	0.47	1.00
6			AT1G62240		CATMA1A51310	8.61	9.16	0.55	1.00
7			AT5G61160		CATMA5A57260	8.58	9.07	0.50	1.00
8			AT2G05440		CATMA2A04270	8.30	11.40	3.10	0.00E+00
9		proline-rich proteins (PRPs)	AT2G21140	AtPRP2	CATMA2A19790	12.47	11.84	-0.63	3.56E-01
10			**AT1G28290**		CATMA1A26460	11.98	11.89	-0.09	1.00
11			AT4G38770	AtPRP4	CATMA4A40195	10.00	9.15	-0.86	7.25E-05
12			AT4G16980		CATMA4A18000	9.07	9.41	0.34	1.00
13			AT4G09530		CATMA5A08360	8.02	9.06	1.04	1.55E-08
14			AT4G38080		CATMA4A39600	7.80	9.32	1.52	0.00E+00
15		leucine-rich-repeat extensin (LRX)	**AT3G24480**	**AtLRX4 (LRR-extensin)**	CATMA3A24360	10.26	10.33	0.08	1.00

1	**Proteins possibly involved in oxido-reduction reactions**	peroxidases	AT2G37130	AtPrx21	CATMA2A35430	14.91	13.66	-1.25	0.00E+00
2			AT4G21960	AtPrx42	CATMA4A23655	13.39	12.71	-0.68	6.22E-02
3			**AT3G21770**	**AtPrx30**	CATMA3B21635	10.92	10.76	-0.16	1.00
4			AT4G33870	AtPrx48	CATMA4A35650	10.83	10.23	-0.60	8.07E-01
5			AT1G05260	AtPrx3	CATMA1A04113	10.78	10.30	-0.47	1.00
6			AT5G64120	AtPrx71	CATMA5A59590	10.52	10.11	-0.41	1.00
7			**AT1G71695**	**AtPrx12**	CATMA1A60876	10.44	9.48	-0.96	7.29E-07
8			**AT5G66390**	**AtPrx72**	CATMA5A61725	9.15	9.38	0.23	1.00
9			AT2G38390	AtPrx23	CATMA2A36690	8.71	9.32	0.61	6.44E-01
10		proteins homologous to berberine bridge enzymes	At5g44380		CATMA5A40150	10.19	9.03	-1.16	2.81E-11
11		proteins homologous to multicopper oxidases	AT5G21105		CATMA5A19660	11.24	11.08	-0.16	1.00
12			AT1G76160	AtSKS5 (homologous to SKU5)	CATMA1A65380	11.24	10.98	-0.26	1.00
13			AT1G41830	AtSKS6 (homologous to SKU5)	CATMA1A35650	10.20	9.74	-0.45	1.00
14			AT4G12420	AtSKU5	CATMA4A12530	9.97	9.70	-0.27	1.00
15			AT4G22010	AtSKS4	CATMA4A23690	9.04	8.47	-0.57	1.00
16			AT5G48450	AtSKS3	CATMA5A44430	9.02	8.66	-0.36	1.00
17		laccases	AT5G60020	AtLAC17	CATMA5A55770	10.33	9.64	-0.69	3.97E-02
18			AT5G03260	AtLAC11	CATMA5A02370	10.29	10.18	-0.11	1.00
19			AT2G38080	AtLAC4 (IRX12) (IRREGULAR XYLEM 12)	CATMA2A36370	9.52	8.80	-0.72	1.40E-02
20		expressed protein (oxido-reductase domain)	AT3G03330		CATMA3A02250	10.86	11.00	0.14	1.00
21		proteins homologous to copper amine oxidase	AT1G62810		CATMA1A51930	9.05	9.27	0.22	1.00
22		phytocyanins (proteins homologous to blue copper binding proteins)	**AT4G12880**	**plastocyanin**	CATMA4A13020	11.56	11.94	0.38	1.00
23			AT2G32300	AtUCC1 (uclacyanin)	CATMA2A30615	11.32	11.29	-0.03	1.00
24			AT5G15350	AtEN22 (plastocyanin domain)	CATMA5A13625	10.22	10.58	0.35	1.00
25			AT1G08500	AtEN21 (plastocyanin domain)	CATMA1A07465	9.67	10.04	0.37	1.00
26			At5G53870	AtEN1 (plastocyanin domain)	CATMA5A49760	9.10	9.24	0.14	1.00
27			AT1G72230	AtUCC8 (uclacyanin)	CATMA1A61460	8.91	9.34	0.43	1.00

1	**Proteins with interacting domains**	proteins homologous to lectins	**AT1G78850**	**curculin-like, mannose binding**	CATMA1A67950	11.87	11.37	-0.50	1.00
2			**AT1G78830**	**curculin-like, mannose-binding**	CATMA1A67930	10.44	9.90	-0.54	1.00
3			AT1G78820	curculin-like, mannose-binding	CATMA1A67920	9.66	8.73	-0.93	3.03E-06
4			AT3G15356	legume lectin	CATMA3A14725	11.58	11.07	-0.51	1.00
5			AT3G54080	legume lectin	CATMA3A47030	9.98	9.92	-0.05	1.00
6			AT1G69295	expressed protein (X8 domain, carbohydrate binding?)	CATMA1A58590	11.17	11.28	0.11	1.00
7			AT1G09460	expressed protein (X8 domain, carbohydrate binding?)	CATMA1A08310	9.09	9.60	0.51	1.00
8			AT5G61130	expressed protein (X8 domain, carbohydrate binding?)	CATMA5A56770	9.04	8.80	-0.24	1.00
9			**AT2G17120**	**expressed protein (peptidoglycan-binding LysM domain)**	CATMA2A15860	9.49	9.27	-0.22	1.00
10		proteins with leucine-rich repeat (LRR) domains	**AT3G20820**	**expressed protein (LRR domains)**	CATMA3A20560	10.84	10.26	-0.59	1.00
11			AT1G28340	expressed protein (LRR domains)	CATMA1A26520	10.25	9.92	-0.33	1.00
12			**AT5G12940**	**expressed protein (LRR domains)**	CATMA5A11170	9.94	9.16	-0.78	1.49E-03
13			AT3G05990	expressed protein (LRR domains)	CATMA3A05050	9.57	9.29	-0.28	1.00
14			AT3G19320	expressed protein (LRR domains)	CATMA3A18956	9.08	9.06	-0.01	1.00
15			AT2G33060	expressed protein (LRR domains)	CATMA2A31230	9.04	8.66	-0.38	1.00
16		enzyme inhibitors	AT3G12880	pectin methylesterase inhibitor	CATMA3A11840	9.41	9.33	-0.08	1.00
17			**AT4G25260**	**pectin methylesterase inhibitor**	CATMA4A26950	9.37	9.86	0.49	1.00
18			**AT3G43270**	**pectin methylesterase inhibitor**	CATMA3A35650	9.30	8.89	-0.41	1.00
19				pectin methylesterase inhibitor	CATMA5A60045	9.00	9.07	0.07	1.00
20			**AT5G06860**	**polygalacturonase inhibiting protein (PGIP1)**	CATMA5A06073	10.78	10.47	-0.30	1.00
21			**AT1G73260**	**serine protease inhibitor**	CATMA1A62540	12.54	13.41	0.86	5.77E-05
22			AT2G31980	protease inhibitor (cystatin)	CATMA2A30250	10.52	10.96	0.44	1.00
23			**AT4G16500**	**protease inhibitor (cystatin)**	CATMA4A17360	9.36	9.60	0.24	1.00
24			AT5G47550	protease inhibitor (cystatin)	CATMA5A43530	8.81	9.18	0.37	1.00
25			AT1G71950	protease inhibitor	CATMA1A61160	9.92	10.36	0.44	1.00
26			AT2G02120	protease inhibitor (gamma-thionin)	CATMA2A01180	9.91	10.89	0.99	2.00E-07
27			AT2G43510	protease inhibitor (ATTI1) (ARABIDOPSIS THALIANA TRYPSIN INHIBITOR PROTEIN 1)	CATMA2A41920	8.40	9.16	0.76	3.69E-03
28			**AT1G17860**	**protease inhibitor (Kunitz)**	CATMA1A16880	9.53	9.73	0.20	1.00
29			AT1G73330	protease inhibitor (Kunitz)	CATMA1A62630	9.22	9.07	-0.15	1.00
30		expressed proteins with diverse structural domains	AT1G21680	expressed protein (WD40-like Beta Propeller Repeat)	CATMA1A20770	11.45	12.08	0.63	3.73E-01
31			AT3G62360	expressed protein (carbohydrate-binding-like fold)	CATMA3A55500	9.14	8.80	-0.34	1.00
32			AT3G51050	expressed protein (FG-GAP repeat that may fold into beta-propeller structure)	CATMA3A44050	10.28	10.00	-0.28	1.00
33			AT3G09090	expressed protein (FG-GAP domain that may fold into beta-propeller structure)	CATMA3A07960	9.60	9.59	-0.02	1.00

1	**Proteins related to lipid metabolism**	proteins homologous to GDSL Lipase/Acylhydrolase	AT3G16370		CATMA3A15780	12.19	11.60	-0.59	1.00
2			AT1G28580		CATMA1A26790	11.62	11.78	0.16	1.00
3			AT2G04570		CATMA2A03550	10.67	9.99	-0.69	4.83E-02
4			AT5G45950		CATMA5A41950	10.61	9.36	-1.25	0.00E+00
5			AT5G45670		CATMA5A41660	10.47	10.56	0.08	1.00
6			AT3G04290		CATMA3A03260	10.15	9.02	-1.13	1.40E-10
7			AT5G03610		CATMA5A02790	9.73	9.60	-0.13	1.00
8			AT1G54000		CATMA1A45100	9.63	8.79	-0.84	1.23E-04
9			AT1G09390		CATMA1A08220	9.33	9.06	-0.28	1.00
10			AT3G14220		CATMA3A13490	9.27	8.59	-0.68	6.28E-02
11			**AT3G48460**		CATMA3A41440	9.03	8.11	-0.91	6.07E-06
12		proteins homologous to plant lipid transfer protein/seed storage/trypsin alpha amylase inhibitor	AT2G38540	LTP1	CATMA2A36820	11.67	11.33	-0.34	1.00
13			AT2G45180		CATMA2A43580	12.81	13.33	0.52	1.00
14			AT1G12090		CATMA1A11135	12.27	12.13	-0.14	1.00
15			AT1G27950		CATMA1A26130	11.15	11.22	0.07	1.00
16			AT3G18280		CATMA3A17820	10.91	12.11	1.20	5.61E-12
17			AT4G22490		CATMA4A24210	10.60	9.66	-0.95	1.47E-06
18			AT3G43720		CATMA3A36600	10.09	10.13	0.04	1.00
19			AT2G15050		CATMA2A13890	10.04	9.92	-0.12	1.00
20			AT3G53980		CATMA3A46940	10.03	10.45	0.42	1.00
21			AT4G15160		CATMA4A15700	9.80	9.31	-0.50	1.00
22			AT1G55260		CATMA1A46320	9.70	9.70	0.01	1.00
23			AT3G22620		CATMA3A22590	9.44	9.61	0.17	1.00
24			AT5G05960		CATMA5A05150	8.64	9.48	0.84	1.29E-04
25			AT2G13820		CATMA2A12440	8.56	9.11	0.55	1.00
26		expressed protein (ML domain - MD-2-related lipid recognition domain)	AT3G44100		CATMA3A36990	10.15	10.63	0.48	1.00
27			AT2G16005		CATMA2A14850	10.08	10.17	0.09	1.00
28		others	AT5G18630	homologous to lipase (class 3)	CATMA5A16940	9.63	9.68	0.05	1.00
29			AT5G14180	homologous to lipase	CATMA5A12400	8.91	10.46	1.55	0.00E+00
30			AT5G62200	homologous to lipase/lipoxygenase	CATMA5A57790	9.56	9.22	0.34	1.00
31			AT3G51730	homologous to saposin	CATMA3A44690	11.12	11.45	0.33	1.00
32			AT5G01800		CATMA5A00860	9.41	9.42	0.02	1.00
33			AT4G26690	homologous to glycerophosphoryl diester phosphodiesterase	CATMA4A28280	9.87	9.58	-0.29	1.00
34			AT4G29700	homologous to type I phosphodiesterase	CATMA4A31330	9.42	8.53	-0.89	1.78E-05
35			AT1G76090	homologous to sterol methyltransferase	CATMA1A65315	11.94	11.93	-0.01	1.00
36			AT1G20330		CATMA1A19326	9.39	9.11	-0.29	1.00
37			**AT5G34850**	**homologous to phosphoesterase**	CATMA5A29650	12.30	11.94	-0.36	1.00
38			AT1G07230		CATMA1A06280	10.08	9.71	-0.36	1.00
39			AT4G01950	homologous to acyltransferase	CATMA4A02220	9.47	8.71	-0.77	2.90E-03
40			AT3G11780	expressed protein (MD-2-related lipid-recognition domain)	CATMA3A10730	10.23	10.33	0.10	1.00E+00

1	**Miscellaneous**		AT3G57010	homologous to strictosidine synthase	CATMA3A50000	10.33	9.69	-0.63	2.89E-01
2			AT3G57030	homologous to strictosidine synthase	CATMA3A50020	9.39	9.37	-0.02	1.00
3			AT3G45040	homologous to cytidylyltransferase	CATMA3A38060	9.90	9.93	0.03	1.00
4			AT5G10480	PEPINO/PASTICCINO2, putative anti-phosphatase (AtPEP/AtPAS2)	CATMA5A09210	9.93	9.33	-0.60	8.58E-01
5			AT5G60920	COBRA	CATMA5A56600	11.44	11.10	-0.34	1.00
6			AT1G03880	homologous to plant seed storage protein (cupin domain)	CATMA1A02730	9.55	9.44	-0.11	1.00
7			**AT2G22170**	**homologous to *Brassica napus* dehydratation stress-induced protein**	CATMA2A20720	11.49	11.29	-0.20	1.00
8			AT3G08650	metal transporter family	CATMA3A07470	9.95	9.57	-0.38	1.00
9			AT5G12200	homologous to amido-hydrolase (metal-dependent hydrolase)	CATMA5A10970	11.15	10.75	-0.41	1.00
10			AT2G34250	homologous to protein transport protein (sec61)	CATMA2A32380	10.93	10.96	0.03	1.00
11			AT4G29720	homologous to flavin containing amine oxidoreductase	CATMA4A31350	9.17	9.15	-0.02	1.00
12			AT3G06550	homologous to Casp1 O-acetyltransferase	CATMA3A05690	9.34	9.07	-0.27	1.00
13			AT4G35220	homologous to cyclase	CATMA4A36950	9.58	9.36	-0.22	1.00
14		proteins homologous to late embryogenesis abundant proteins	AT3G17520		CATMA3A16945	8.53	9.32	0.79	1.10E-03
15			AT2G41260	M17	CATMA2A39610	8.99	9.69	0.70	3.02E-02
16		proteins homologous to dirigent proteins	AT2G28670		CATMA2A27080	11.91	11.37	-0.55	1.00
17			AT4G23690		CATMA4A25520	9.59	9.63	0.04	1.00
18		hormone-related proteins	AT2G17500	homologous to auxin efflux carrier	CATMA2B16180	10.92	10.66	-0.25	1.00
19			AT1G68100	protein required for auxin conjugate sensitivity (AtIAR 1) (Zip Zinc transporter domain)	CATMA1A57475	9.72	9.64	-0.09	1.00
20			**AT5G15230**	**gibberellin regulated protein (GASA4)**	CATMA5A13515	13.16	13.93	0.77	2.75E-03
21			AT2G14900	gibberellin regulated protein (GASA)	CATMA2A13660	10.34	11.23	0.89	1.82E-05
22			AT1G74670	gibberellin regulated protein (GASA)	CATMA1A64040	8.31	9.84	1.53	0.00E+00
23		pathogenesis-related proteins	AT3G04720	pathogenesis-related protein (AtPR4) (Carbohydrate Binding-Module family 18 - CBM18)	CATMA3A03743	10.57	10.58	0.00	1.00
24			AT1G75800	homologous to thaumatin	CATMA1A65090	10.97	10.73	-0.24	1.00
25			AT5G24620	homologous to thaumatin	CATMA5A22250	9.62	9.43	-0.19	1.00
26			AT4G36010	homologous to thaumatin	CATMA4A37660	9.21	9.54	0.34	1.00
27			AT1G18520	homologous to thaumatin	CATMA1A17240	9.20	9.44	0.25	1.00
28		proteins homologous to germin	AT5G20630	AtGER3	CATMA5A19130	12.46	11.16	-1.30	0.00
29			**AT1G09560**	**GLP5 (subfamily 2, member 1)**	CATMA1A08413	11.34	11.06	-0.28	1.00
30			AT5G03460	GLP1	CATMA5A02640	10.72	11.43	0.71	2.07E-02
31			AT3G10080		CATMA3A08960	9.56	9.44	-0.12	1.00
32		proteins homologous to phosphate-induced proteins (phi)	**AT5G09440**		CATMA5A08270	12.68	12.14	-0.54	1.00
33			AT5G51550		CATMA5A47480	9.83	10.20	0.36	1.00
34			AT4G08950		CATMA4A08890	10.87	9.93	-0.94	1.69E-06
35		proteins homologous to acid phosphatases	**AT2G27190**		CATMA2A25560	11.34	11.09	-0.25	1.00
36			AT5G44020		CATMA5A39790	10.62	10.58	-0.05	1.00
37			AT2G01890	AtPAP8	CATMA2A00940	10.29	10.45	0.16	1.00
38			AT1G25230	AtPAP3	CATMA1A23880	11.05	10.37	-0.67	7.49E-02
39			AT1G13900		CATMA1A12860	9.28	8.99	-0.28	1.00
40			AT3G20500		CATMA3A20210	9.15	9.32	0.18	1.00
41		proteins homologous to ribonucleases	AT1G24450		CATMA1A23390	9.33	9.85	0.52	1.00
42			AT2G39780	RNS2	CATMA2A38006	9.73	9.88	0.15	1.00
43			AT1G26820	RNS3	CATMA1A25040	8.82	9.42	0.60	8.50E-01

1	**Unknown function**	expressed proteins	AT5G39570		CATMA5A35150	12.21	11.93	-0.27	1.00
2			AT3G13410		CATMA3A12540	11.85	11.55	-0.30	1.00
3			AT5G20520		CATMA5A19000	11.64	12.17	0.54	1.00
4			AT3G15950		CATMA3A15360	11.44	10.99	-0.45	1.00
5			AT1G78040		CATMA1A67150	11.39	11.42	0.03	1.00
6			AT3G24160		CATMA3A24093	11.28	11.06	-0.21	1.00
7			AT5G25265		CATMA5A22980	11.02	10.80	-0.22	1.00
8			AT5G52180		CATMA5A48120	10.83	10.48	-0.35	1.00
9			AT1G65720		CATMA1A55000	10.78	11.48	0.70	2.69E-02
10			AT5G58375		CATMA5A54130	10.66	11.41	0.75	5.56E-03
11			AT4G39840		CATMA4A41230	10.59	10.39	-0.20	1.00
12			AT5G17190		CATMA5A15470	10.43	10.94	0.51	1.00
13			AT2G42840		CATMA2A41257	10.42	9.80	-0.63	3.54E-01
14			AT1G61900		CATMA1A50970	10.18	10.00	-0.18	1.00
15			AT1G43580		CATMA1A36910	10.17	10.40	0.24	1.00
16			AT2G12400		CATMA2A10420	10.15	9.64	-0.50	1.00
17			AT5G61820		CATMA5A57420	10.13	10.38	0.25	1.00
18			AT4G27120		CATMA4A28730	10.12	10.10	-0.03	1.00
19			AT5G13140		CATMA5A11350	10.09	9.61	-0.48	1.00
20			AT1G69980		CATMA1A59280	10.04	10.44	0.40	1.00
21			AT1G47310		CATMA1A38230	10.03	9.89	-0.13	1.00
22			AT1G48440		CATMA1A39540	9.88	10.25	0.36	1.00
23			AT1G61150		CATMA1A50200	9.83	9.74	-0.09	1.00
24			AT3G09570		CATMA3A08440	9.77	9.72	-0.04	1.00
25			AT3G27390		CATMA3A27250	9.76	9.20	-0.57	1.00
26			AT1G67785		CATMA1A57160	9.74	10.42	0.67	7.67E-02
27			AT3G51580		CATMA3A44590	9.65	9.55	-0.10	1.00
28			AT5G56170		CATMA5A51950	9.63	9.94	0.30	1.00
29			**AT2G04690**		CATMA2A03640	9.61	9.55	-0.06	1.00
30			AT3G14920		CATMA3A14280	9.54	9.12	-0.41	1.00
31			AT4G30450		CATMA4A32060	9.48	9.94	0.46	1.00
32			AT1G29790		CATMA1A27760	9.47	9.28	-0.20	1.00
33			AT3G18050		CATMA3A17520	9.43	9.28	-0.15	1.00
34			AT5G01075		CATMA5A00100	9.34	9.58	0.25	1.00
35			AT2G12845		CATMA1A11840	9.26	9.21	-0.05	1.00
36			AT4G16515		CATMA4A17380	9.20	9.76	0.55	1.00
37			AT1G77350		CATMA1A66570	9.17	9.54	0.37	1.00
38			AT2G42390		CATMA2A40790	9.16	9.51	0.35	1.00
39			AT1G28400		CATMA1A26600	9.16	9.30	0.15	1.00
40			AT1G80860		CATMA1A70080	9.06	9.42	0.36	1.00
41			AT3G62580		CATMA3A55750	9.07	8.85	-0.22	1.00
42			AT5G38980		CATMA5A34590	9.07	9.10	0.03	1.00
43			AT5G58640		CATMA5A54390	9.02	9.26	0.24	1.00
44			AT2G35850		CATMA2B34030	8.98	9.30	0.32	1.00
45			AT2G27385		CATMA2A25790	8.97	9.44	0.47	1.00
46			AT3G52360		CATMA3A45300	8.96	9.06	0.10	1.00
47			AT2G31090		CATMA2A29310	8.89	9.39	0.50	1.00
48			AT2G32380		CATMA2A30680	8.92	9.37	0.46	1.00
49			AT1G53650		CATMA5A49530	8.74	9.74	1.00	1.07E-07
50			AT5G14105		CATMA5A12330	8.67	9.36	0.69	4.57E-02
51			AT1G78895		CATMA1A68000	8.49	9.10	0.61	6.72E-01
52			AT5G18850		CATMA5A17220	8.38	9.27	0.89	2.02E-05
53			AT5G40960		CATMA5A36630	7.57	9.10	1.54	0.00E+00
54		expressed proteins with domains of unknown function (DUF) or uncharacterized protein families (UPF)	AT4G01450	DUF6	CATMA4A01650	9.76	9.43	-0.34	1.00
55			AT4G28050	DUF6	CATMA3A27840	9.50	9.46	-0.04	1.00
56			AT1G61740	DUF81	CATMA1A50820	10.12	10.37	0.25	1.00
57			AT3G01100	DUF221	CATMA3A00135	9.90	9.85	-0.05	1.00
58			AT1G30360	DUF221	CATMA1A28370	11.51	10.82	-0.69	4.64E-02
59			AT2G30900	DUF231	CATMA2A29100	9.93	9.73	-0.19	1.00
60			AT3G54260	DUF231	CATMA3A47210	8.58	9.75	1.17	1.12E-11
61			AT1G51630	DUF246	CATMA1A42760	10.35	9.79	-0.56	1.00
62			AT3G23300	DUF248	CATMA3A23300	10.42	10.01	-0.41	1.00
63			AT3G17430	DUF250	CATMA3A16860	9.58	9.45	-0.13	1.00
64			AT2G28315	DUF250	CATMA2A26710	9.10	8.77	-0.33	1.00
65			AT5G26740	DUF300	CATMA5A24080	10.67	10.74	0.06	1.00
66			AT5G19590	DUF538	CATMA5A18020	10.66	10.72	0.06	1.00
67			AT5G19860	DUF538	CATMA5A18290	11.13	10.86	-0.27	1.00
68			AT4G02370	DUF538	CATMA4A02670	11.18	11.56	0.39	1.00
69			AT3G07460	DUF538	CATMA3A06690	10.35	10.48	0.13	1.00
70			AT1G02816	DUF538	CATMA1A01750	8.87	9.00	0.14	1.00
71			AT3G25290	DUF568	CATMA3A25140	9.33	9.15	-0.17	1.00
72			AT2G04850	DUF568	CATMA2A03790	9.07	8.69	-0.38	1.00
73			AT1G27930	DUF579	CATMA1A26110	9.91	9.97	0.06	1.00
74			AT4G25830	DUF588	CATMA4A27500	9.19	8.96	-0.23	1.00
75			AT1G08800	DUF593	CATMA1A07710	9.02	9.28	0.25	1.00
76			**AT4G32460**	**DUF642**	CATMA4A34200	10.87	10.10	-0.77	2.20E-03
77			**AT5G25460**	**DUF642**	CATMA5A23220	9.86	9.42	-0.43	1.00
78			AT1G29980	DUF642	CATMA1A28000	10.14	9.74	-0.40	1.00
79			**AT3G08030**	**DUF642**	CATMA3A07290	12.40	12.06	-0.35	1.00
80			**AT5G11420**	**DUF642**	CATMA5A10190	12.14	12.05	-0.09	1.00
81			AT1G55240	DUF716	CATMA1A46290	8.98	10.06	1.08	0.00
82			AT4G04990	DUF761	CATMA4A05610	9.33	9.33	0.00	1.00
83			AT4G49310	DUF791	CATMA3A42340	9.01	8.78	-0.23	1.00
84			AT4G00860	DUF1138	CATMA4A00935	9.32	9.77	0.45	1.00
85			AT3G15480	DUF1218	CATMA3A14890	11.10	11.15	0.06	1.00
86			AT4G31130	DUF1218	CATMA4A32820	9.32	9.33	0.01	1.00
87			AT1G68220	DUF1218	CATMA1A57600	8.74	9.16	0.42	1.00
88			AT1G30130	DUF1365	CATMA1A28150	10.06	9.91	-0.15	1.00
89			AT1G27000	DUF1664	CATMA1A25200	10.79	10.76	-0.03	1.00
90			AT4G24330	DUF1682	CATMA4A26080	9.66	9.43	-0.24	1.00
91			AT4G30660	UPF0057	CATMA4A32270	8.84	9.47	0.63	3.58E-01
92			AT2G24040	UPF0057	CATMA2A22390	8.80	9.51	0.71	2.34E-02
93			AT5G36290	UPF0016	CATMA5A31840	9.70	9.77	0.08	1.00
94			AT2G20510	UPF0136	CATMA3A20220	10.05	10.60	0.55	1.00
95			AT1G36980	UPF0220	CATMA1A34890	8.88	9.22	0.34	1.00
96		uncharacterized conserved protein	AT1G57680	UCP031277	CATMA1A47970	11.34	10.99	-0.35	1.00

97		expressed proteins with diverse structural domains	AT1G07080	GILT domain	CATMA1A06150	11.44	11.49	0.05	1.00
98			AT5G25610	BURP domain	CATMA5A23380	13.08	12.33	-0.76	4.22E-03
99			**AT3G20370**	**MATH domain**	CATMA3A20050	11.42	10.92	-0.50	1.00
100			**AT3G22640**	**cupin domain**	CATMA3A22600	8.53	9.00	0.47	1.00
101			AT1G71980	PA domain (Protease-Associated)	CATMA1A61200	9.43	8.91	-0.52	1.00
102			**AT2G28490**	**cupin domain**	CATMA2A26920	9.21	9.43	0.22	1.00
103			AT1G53210	calcium-binding EF-hand domains	CATMA1A44240	11.05	10.58	-0.47	1.00
104			AT1G29020	calcium-binding EF-hand domains	CATMA1A26980	10.36	10.09	-0.27	1.00
105			AT4G27790	calcium-binding EF-hand domains	CATMA4A29420	10.76	10.72	-0.04	1.00
106			AT2G20990	calcium dependent membrane targeting domain, CaLB protein	CATMA2A19620	10.52	10.20	-0.31	1.00
107			AT1G05500	calcium dependent membrane targeting domain, CaLB protein	CATMA1A04400	10.25	9.82	-0.43	1.00
108			AT4G04800	SelR domain	CATMA4A05380	8.94	9.45	0.51	1.00
109			AT3G61050	calcium dependent membrane targeting domain, CaLB protein	CATMA3A54220	10.65	10.18	-0.47	1.00
110			AT1G53590	calcium dependent membrane targeting domain, CaLB protein	CATMA1A44600	9.22	9.00	-0.22	1.00
111			AT1G21670	WD40-like Beta Propeller Repeat	CATMA1A20760	9.61	9.68	0.07	1.00
112			AT3G08930	integral membrane protein	CATMA3A07760	11.29	11.00	-0.28	1.00
113			AT5G18520	integral membrane protein	CATMA5A16820	11.63	11.34	-0.29	1.00
114			AT5G42090	integral membrane protein	CATMA5A37800	10.50	10.12	-0.38	1.00
115			AT5G01460	integral membrane protein	CATMA5A00520	9.72	9.59	-0.13	1.00
116			AT1G61670	transmembrane receptor domain	CATMA1A50770	9.09	8.87	-0.22	1.00
117			AT4G39730	membrane or lipid associated protein domain	CATMA4A41080	11.30	10.78	-0.52	1.00
118			AT2G25110	MIR domain	CATMA2A23430	9.35	9.78	0.43	1.00
119			AT5G40480	invasin/intimin domain	CATMA5A36140	9.01	9.32	0.30	1.00
120			AT5G25100	EMP70 domain	CATMA5A22810	9.83	9.70	-0.13	1.00
121			AT1G64670	alpha/beta hydrolase fold	CATMA1A53990	10.57	9.78	-0.78	1.46E-03
122		other expressed proteins	AT4G14420	protein associated to hypersensitive response	CATMA4A14790	9.87	10.32	0.45	1.00
123			AT5G43460	protein associated to hypersensitive response	CATMA5A39320	9.74	10.56	0.83	2.63E-04
124			AT2G41470	embryo specific protein	CATMA2A39880	10.43	10.84	0.41	1.00
125			**AT2G34700**	**pollen proteins Ole e I family**	CATMA2A32820	11.53	9.27	-2.27	0.00E+00
