# Supplementary material for: Cell wall biogenesis of Arabidopsis thaliana elongating cells: transcriptomics complements proteomics
Source: BMC Genomics. 2009 Oct 31;10:505. doi: 10.1186/1471-2164-10-505 (PMC2774874; doi:10.1186/1471-2164-10-505)
Supplement: Additional file 4 — Secretory Pathway Genes (SPGs) with modulated levels of transcripts in 5- and 11-day-old etiolated hypocotyls. The data provided represent the transcript levels of SPGs differentially expressed between the two samples. [file 1471-2164-10-505-S4.pdf]

Additional file 4. Secretory Pathway Genes (SPGs) with modulated levels of transcripts in 5- and 11-day-old hypocotyls. SPGs were annotated using bioinformatic software for sub-cellular localization and functional domains as described in Methods. The intensity of the signal is expressed as $\log_2$, the $\log_2$ of ratio between the levels of transcripts at 11-days and 5-days as well as the p-value are indicated (Bonferroni p-value<5%).

Functional class	Gene family	AGI number	gene name	5-day-old hypocotyls	11-day-old hypocotyls	$\log_2$ of ratio 11-days / 5-days	p-value
**Level of transcripts higher in 5-day-old than in 11-day-old hypocotyls**					$\log_2$		
**Proteins involved in polysaccharide synthesis**	glycosyl transferase family 2 (cellulose synthases)	AT5G09870	AtCESA5	10.43	9.65	-0.78	1.64E-03
	glycosyl transferase family 48 (callose synthases)	AT1G05570	AtCALS1	9.77	9.06	-0.72	1.84E-02
**Proteins acting on carbohydrates**	glycoside hydrolase family 1 (beta-glucosidase)	AT3G60130		11.42	10.59	-0.83	2.39E-04
		AT5G26000		10.15	9.39	-0.76	4.17E-03
	glycoside hydrolase family 9	AT1G64390		11.38	10.66	-0.72	1.41E-02
	glycoside hydrolase family 16	AT4G14130	AtXTH15	13.61	12.31	-1.31	0.00E+00
		AT2G36870	AtXTH32	8.82	7.91	-0.91	6.19E-06
		AT3G44990	AtXTH31	10.10	8.76	-1.35	0.00E+00
	glycoside hydrolase family 17	AT3G04010		10.25	9.24	-1.01	6.09E-08
	glycoside hydrolase family 19	AT2G43590		9.61	8.31	-1.30	0.00E+00
	carbohydrate esterase family 8 (pectin methylesterases)	AT3G49220		11.84	11.15	-0.69	3.94E-02
		AT2G43050		12.72	11.24	-1.48	0.00E+00
		AT3G14310	AtPME3	11.66	10.82	-0.84	1.44E-04
**Proteins possibly involved in oxido-reduction reactions**	peroxidases	AT2G37130	AtPrx21	14.91	13.66	-1.25	0.00E+00
		AT1G71695	AtPrx12	10.44	9.48	-0.96	7.29E-07
		AT5G17820	AtPrx57	8.01	7.07	-0.94	2.37E-06
	laccases	AT5G60020	AtLAC17	10.33	9.64	-0.69	3.97E-02
		AT2G38080	AtLAC4 (IRX12)	9.52	8.80	-0.72	1.40E-02
	proteins homologous to berberine bridge enzymes	AT5G44380		10.19	9.03	-1.16	2.81E-11
**Proteases**	serine proteases (proteins homologous to serine proteases)	AT5G51750	AtSBT1.3	11.18	9.81	-1.37	0.00E+00
		AT3G14067	AtSBT1.4	12.34	11.18	-1.16	2.25E-11
		At2G05920	AtSBT1.8	12.30	11.50	-0.81	6.10E-04
		AT5G59090	AtSBT4.12	11.25	10.12	-1.13	1.46E-10
		AT2G04160	AtSBT5.3 (AIR3)	8.53	7.61	-0.92	3.86E-06
	cysteine proteases	AT2G34080		7.97	7.13	-0.85	1.16E-04
		AT3G43960		9.18	8.40	-0.78	1.71E-03
		AT4G36880		9.40	8.26	-1.14	6.74E-11
	aspartyl proteases	AT1G66180		11.80	11.06	-0.74	7.96E-03
		AT3G52500		9.46	8.75	-0.72	1.79E-02
		AT5G10770		11.73	9.55	-2.17	0.00E+00
	peptidases	AT4G32940	asparginyl peptidase	12.75	11.82	-0.93	3.04E-06
		AT4G38220	metallopeptidases M20/M25/M40	10.76	9.95	-0.80	7.27E-04
**Proteins possibly involved in signaling**	proteins homologous to receptor-kinases (RLKs)	AT1G08590	CLAVATA1	9.60	8.76	-0.85	1.12E-04
		AT3G49670	BAM2	10.09	9.39	-0.70	3.09E-02
		AT2G26330	ERECTA	9.90	8.99	-0.90	9.91E-06
**Structural proteins**	glycine-rich proteins (GRPs)	AT1G04800		8.56	7.82	-0.73	1.03E-02
	proline-rich proteins (GRPs)	AT4G38770	AtPRP4	10.00	9.15	-0.86	7.25E-05

**Proteins with interacting domains**	proteins homologous to lectins	AT1G78820	curculin-like, mannose-binding	9.66	8.73	-0.93	3.03E-06
		AT5G18470		8.98	8.28	-0.69	3.84E-02
	enzyme inhibitors	AT1G72290	protease inhibitor (Kunitz)	8.51	7.56	-0.95	1.31E-06
	LRR proteins	AT5G12940		9.94	9.16	-0.78	1.49E-03
	proteins with fasciclin domains	AT3G52370	expressed protein	9.18	8.39	-0.79	1.22E-03
**Proteins related to lipid metabolism**	proteins homologous to GDSL Lipase/Acylhydrolase	AT5G45950		10.61	9.36	-1.25	0.00E+00
		AT1G54000		9.63	8.79	-0.84	1.23E-04
		AT2G04570		10.67	9.99	-0.69	4.83E-02
		AT2G42990		8.99	8.14	-0.85	1.09E-04
		AT3G04290	AtLTL1	10.15	9.02	-1.13	1.40E-10
		AT5G55050		8.50	7.77	-0.72	1.32E-02
		AT4G30140		8.09	7.06	-1.04	1.74E-08
		AT3G48460		9.03	8.11	-0.91	6.07E-06
	proteins homologous to plant lipid transfer protein/seed storage/trypsin alpha amylase inhibitor	AT4G22490		10.60	9.66	-0.95	1.47E-06
	proteins homologous to acyltransferase	AT4G01950		9.47	8.71	-0.77	2.90E-03
	alpha/beta-hydrolase fold protein superfamily	AT1G64670	BODYGUARD	10.57	9.78	-0.78	1.46E-03
	protein homologous to type I phosphodiesterase	AT4G29700		9.42	8.53	-0.89	1.78E-05
**Miscellaneous**		AT4G08950	homologous to *N. tabacum* phi proteins (EXORDIUM)	10.87	9.93	-0.94	1.69E-06
		AT5G25610	RD22 RESPONSIVE TO DESSICATION 22) (BURP domain)	13.08	12.33	-0.76	4.22E-03
		AT1G19900	homologous to glyoxal oxidase and DUF1929	7.85	6.96	-0.89	1.50E-05
		AT3G05950	homologous to germin	6.72	7.95	1.23	0.00E+00
		AT5G20630	homologous to germin (AtGLP3)	12.46	11.16	-1.30	0.00E+0
**Unknown function**	expressed proteins with domains of unknown function (DUF) or uncharacterized protein families (UPF)	AT1G30360	DUF221	11.51	10.82	-0.69	4.64E-02
		AT4G32460	DUF642	10.87	10.10	-0.77	2.20E-03
	other expressed proteins	AT2G34700	homolog to *N. tabacum* pistil-specific protein	11.53	9.27	-2.27	0.00E+00
		AT2G32560		9.65	8.69	-0.95	9.55E-07

**Level of transcripts higher in 11-day-old than in 5-day-old hypocotyls**							
**Proteins acting on carbohydrates**	glycoside hydrolase family 1 (beta-glucosidase)	AT3G60140		8.01	10.03	2.01	0.00E+00
**Proteins possibly involved in oxido-reduction reactions**	phytocyanins (proteins homologous to blue copper binding proteins)	AT5G20230	plastocyanin (AtSTC1)	7.32	8.73	1.41	0.00E+00
**Proteases**	cysteine proteases (papain family)	AT3G54940		7.81	8.75	0.94	1.90E-06
**Proteins possibly involved in signaling**	arabinogalactan proteins (AGPs)	AT4G09030	AtAGP10	8.18	8.88	0.70	2.80E-02
		AT3G13520	AtAGP12	11.93	12.97	1.04	1.23E-08
		AT3G61640	AtAGP20	9.91	10.65	0.74	6.96E-03
		AT5G53250	AtAGP22	9.98	10.94	0.96	6.83E-07
	proteins homologous to receptor-kinases (RLKs)	AT4G27290		6.80	7.73	0.92	3.96E-06
**Structural proteins**	glycine-rich proteins (GRPs)	AT2G05440		8.30	11.40	3.10	0.00E+00
		AT2G05540		12.45	13.37	0.92	3.93E-06
	proline-rich proteins (PRPs)	AT5G09520		6.89	7.75	0.87	4.91E-05
		AT5G05930		8.02	9.06	1.04	1.55E-08
**Proteins with interacting domains**	enzyme inhibitors	AT1G75830	protease inhibitor (gamma-thionin)	8.19	8.90	0.71	2.01E-02
		AT2G02120	protease inhibitor (gamma-thionin)	9.91	10.89	0.99	2.00E-07
		AT2G43510	protease inhibitor (trypsin inhibitor)	8.40	9.16	0.76	3.69E-03
		AT1G73260	protease inhibitor (Kunitz)	12.54	13.41	0.86	5.77E-05
**Proteins related to lipid metabolism**	proteins homologous to plant lipid transfer protein/seed storage/trypsin alpha amylase inhibitor	AT3G18280		10.91	12.11	1.20	5.61E-12
		AT5G05960		8.64	9.48	0.84	1.29E-04
	others	AT5G14180	homolog to lipase	8.91	10.46	1.55	0.00E+00
**Miscellaneous**	proteins homologous to late embryogenesis abundant proteins	AT3G17520		8.53	9.32	0.79	1.10E-03
		AT2G41260	M17	8.99	9.69	0.70	3.02E-02
	hormone-related proteins	AT1G04560	homolog to AWPM (ABA-induced)	7.81	8.97	1.16	2.25E-11
		AT1G74670	gibberellin regulated protein (GASA)	8.31	9.84	1.53	0.00E+00
		At2G14900	gibberellin regulated protein (GASA)	10.34	11.23	0.89	1.82E-05
		AT3G02885	gibberellin regulated protein (GASA5)	7.22	7.90	0.69	4.85E-02
		AT5G15230	gibberellin regulated protein (GASA4)	13.16	13.93	0.77	2.75E-03
		AT4G09600	gibberellin regulated protein (GASA3)	7.78	8.61	0.83	2.53E-04
	others	AT2G22860	phytosulfokine (AtPSK2)	8.57	9.60	1.03	2.09E-08
		AT3G49780	phytosulfokine (AtPSK4)	9.00	10.13	1.13	1.01E-10
**Unknown function**	expressed proteins	AT5G40960		9.10	7.57	1.54	0.00E+00
		AT1G65720		10.78	11.48	0.70	2.69E-02
		AT2G31945		6.97	7.69	0.72	1.76E-02
		AT3G45160		7.47	8.28	0.81	4.96E-04
		AT4G38080		9.32	7.80	1.52	0.00E+00
		AT5G03460		10.72	11.43	0.71	2.07E-02
		AT5G14150		8.67	9.36	0.69	4.57E-02
		AT5G18850		8.38	9.27	0.89	2.02E-05
		AT5G58375		10.66	11.41	0.75	5.56E-03
		AT5G43460		9.74	10.56	0.83	2.63E-04
		AT5G64510		8.05	7.29	0.75	4.42E-03

		AT5G53650		9.74	8.74	1.00	1.07E-07
	expressed proteins with domains of unknown function (DUF) or uncharacterized protein families (UPF)	AT3G54260	DUF231	9.75	8.58	1.17	1.12E-11
		AT1G55240	DUF716	8.98	10.06	1.08	2.10E-09
		AT2G24040	UPF0057	8.80	9.51	0.71	2.34E-02
	expressed proteins with diverse structural domains	AT1G49320	BURP domain	8.87	7.80	1.07	3.79E-09
