# Supplementary material for: Cell wall biogenesis of Arabidopsis thaliana elongating cells: transcriptomics complements proteomics
Source: BMC Genomics. 2009 Oct 31;10:505. doi: 10.1186/1471-2164-10-505 (PMC2774874; doi:10.1186/1471-2164-10-505)
Supplement: Additional file 5 — Proteins extracted and identified by mass spectrometry from purified cell walls of 5- and 11-day-old etiolated hypocotyls of A. thaliana. The data provided represent the levels of transcripts of the genes encoding the proteins identified by proteomics. [file 1471-2164-10-505-S5.pdf]

Additional file 5. Proteins extracted and identified by mass spectrometry from purified cell walls of 5- and 11-day-old etiolated hypocotyls of *A. thaliana*. All these proteins were annotated using bioinformatic softwares for sub-cellular localization and functional domains as described in Material and methods. Proteomic data are in Irshad *et al.* (2008). Presence of proteins at 5-days or 11-days is indicated by a ticked box. For transcriptomics, the intensity of the signal is expressed as log_2, the ratio between the levels of transcripts at 11-days and 5-days as well as the p-value are indicated. Background level for transcriptomics is 6.83 (grey boxes indicate results below background). Genes encoding proteins with predicted GPI anchors or trans-membrane domains are in bold, and in pink boxes respectively.

		proteomic data		transcriptomic data			
**accession AGI**	**annotation**	**5-days**	**11-days**	**5-days**	**11-days**	**log_2 of ratio 11-days/5-days**	**p-value**
**proteins acting on carbohydrates**							
At2g06850	glycoside hydrolase family 16 (endoxyloglucan transferase) (AtXTH4)	✓	✓	13.27	12.62	-0.65	1.96E-01
At5g13870	glycoside hydrolase family 16 (endoxyloglucan transferase) (AtXTH5)	✓		9.02	8.63	-0.39	1.00
At3g44990	glycoside hydrolase family 16 (endoxyloglucan transferase) (AtXTH31)	✓		nd	nd		
At2g36870	glycoside hydrolase family 16 (endoxyloglucan transferase) (AtXTH32)		✓	8.82	7.91	-0.91	6.19E-06
At1g10550	glycoside hydrolase family 16 (endoxyloglucan transferase) (AtXTH33)	✓	✓	6.47	6.47	0.00	1.00
At4g16260	glycoside hydrolase family 17		✓	7.24	7.30	0.05	1.00
At4g19810	glycoside hydrolase family 18 (chitinase)	✓		7.19	6.84	-0.35	1.00
At3g12500	glycoside hydrolase family 19 (chitinase)	✓	✓	8.43	8.11	-0.32	1.00
At3g55260	glycoside hydrolase family 20 (beta-hexosaminidase)	✓		10.40	9.90	-0.51	1.00
At1g19170	glycoside hydrolase family 28 (polygalacturonase)	✓		6.92	6.90	-0.03	1.00
At2g33160	glycoside hydrolase family 28 (polygalacturonase)	✓		6.60	6.62	0.02	1.00
At3g06770	glycoside hydrolase family 28 (polygalacturonase)		✓	9.51	9.32	-0.19	1.00
At3g16850	glycoside hydrolase family 28 (polygalacturonase)	✓	✓	8.39	8.18	-0.20	1.00
At3g61490	glycoside hydrolase family 28 (polygalacturonase)	✓	✓	8.91	8.80	-0.11	1.00
At4g18180	glycoside hydrolase family 28 (polygalacturonase)	✓		6.73	6.75	0.02	1.00
At1g68560	glycoside hydrolase family 31 (alpha-xylosidase) (AtXYL1)	✓	✓	10.97	10.57	-0.41	1.00
At3g13790	glycoside hydrolase family 32 (beta-fructofuranosidase)	✓	✓	6.50	6.60	0.09	1.00
At5g34940	glycoside hydrolase family 79 (endo beta-glucuronidase/heparanase)	✓	✓	7.18	7.09	-0.09	1.00
At1g11580	carbohydrate esterase family 8 (pectin methylesterase)	✓		7.65	7.31	-0.34	1.00
**At1g53830**	carbohydrate esterase family 8 (pectin methylesterase) (AtPME2)	✓	✓	9.01	8.39	-0.62	5.17E-01
At3g14310	carbohydrate esterase family 8 (pectin methylesterase) (AtPME3)	✓	✓	11.66	10.82	-0.84	1.44E-04
At3g43270	carbohydrate esterase family 8 (pectin methylesterase)	✓	✓	9.30	8.89	-0.41	1.00
At4g33220	carbohydrate esterase family 8 (pectin methylesterase)	✓	✓	8.59	8.11	-0.48	1.00
**At5g53370**	carbohydrate esterase family 8 (pectin methylesterase)	✓		7.30	7.03	-0.27	1.00
At4g37950	polysaccharide lyase family 4 (rhamnogalacturonate lyase)		✓	6.64	6.57	-0.06	1.00
At1g05570	glycosyl transferase family 48 (callose synthase) (AtCalS1)	✓	✓	9.77	9.06	-0.72	1.84E-02
At5g02260	alpha-expansin (AtEXPA9)	✓	✓	12.95	12.54	-0.41	1.00
At1g20190	alpha-expansin (AtEXPA11)	✓	✓	9.56	9.31	-0.24	1.00
At5g39270	alpha expansin (AtEXP22)	✓		nd	nd		
At3g45970	expansin-like A (AtEXLA1)	✓	✓	nd	nd		
At4g38400	expansin-like A (AtEXLA2)	✓	✓	8.32	8.09	-0.23	1.00
At3g45960	expansin-like A (AtEXLA3)	✓		7.67	7.64	-0.03	1.00

**oxido-reductases**							
At1g71695	peroxidase (AtPrx12)	✓	✓	10.44	9.48	-0.96	7.29E-07
At3g21770	peroxidase (AtPrx30)	✓	✓	10.39	10.30	-0.08	1.00
At3g32980	peroxidase (AtPrx32)	✓	✓	nd	nd		
At3g49110	peroxidase (AtPrx33)		✓	6.37	6.28	0.09	1.00
At3g49120	peroxidase (AtPrx34)	✓	✓	nd	nd		
At3g50990	peroxidase (AtPrx36)	✓		6.53	6.65	0.11	1.00
At4g25980	peroxidase (AtPrx43)		✓	6.52	6.58	0.05	1.00
At4g30170	peroxidase (AtPrx45)	✓	✓	7.91	7.75	-0.16	1.00
At5g17820	peroxidase (AtPrx57)	✓		8.01	7.07	-0.94	2.37E-06
At5g64100	peroxidase (AtPrx69)	✓	✓	7.42	6.80	-0.63	3.63E-01
At5g66390	peroxidase (AtPrx72)		✓	9.15	9.38	0.23	1.00
At2g30210	homologous to laccase (AtLAC3)	✓	✓	8.40	8.42	0.01	1.00
At1g30710	homologous to berberine-bridge enzyme (S)-reticulin:oxygen oxidoreductase	✓		6.48	6.59	0.11	1.00
At4g20860	homologous to berberine-bridge enzyme (S)-reticulin:oxygen oxidoreductase		✓	7.71	7.80	0.09	1.00
At5g44360	homologous to berberine-bridge enzyme (S)-reticulin:oxygen oxidoreductase	✓	✓	6.50	6.59	0.09	1.00
At5g44410	homologous to berberine-bridge enzyme (S)-reticulin:oxygen oxidoreductase		✓	6.69	6.81	0.12	1.00
At1g01980	homologous to berberine-bridge enzyme (S)-reticulin:oxygen oxidoreductase		✓	6.50	6.48	-0.01	1.00
At5g22140	expressed protein (oxido-reductase domain)	✓		nd	nd		
At5g56490	expressed protein (FAD binding domain)	✓		nd	nd		
At2g02850	plantacyanin ARPN (blue copper binding protein)		✓	7.26	7.22	-0.04	1.00
At4g12880	early nodulin AtEN20 homologous to blue copper binding protein	✓	✓	11.56	11.94	0.38	1.00
**proteins with interacting domains**							
At1g53070	homologous to lectin (legume lectin domains)	✓	✓	8.45	8.11	-0.34	1.00
At1g78820	homologous to lectin (curculin-like)	✓		9.66	8.73	-0.93	3.03E-06
At1g78830	homologous to lectin (curculin-like)	✓	✓	10.44	9.90	-0.54	1.00
At1g78850	homologous to lectin (curculin-like)	✓	✓	11.87	11.37	-0.50	1.00
At1g78860	homologous to lectin (curculin-like)	✓	✓	7.53	7.99	0.45	1.00
At5g06860	PGIP1 (LRR domains)	✓	✓	nd	nd		
T23B7.10	homologous to PGIP1 (LRR protein FLR1)	✓	✓	nd	nd		
At5g12940	homologous to *Phaseolus vulgaris* PGIP2 (LRR domains)	✓	✓	9.94	9.16	-0.78	1.49E-03
At1g33590	expressed protein (LRR domains)	✓	✓	nd	nd		
At2g34930	expressed protein (LRR domains)	✓	✓	7.43	7.45	0.02	1.00
At3g20820	expressed protein (LRR domains)	✓	✓	10.84	10.26	-0.59	1.00
**At2g17120**	expressed protein (LysM domain)	✓	✓	9.49	9.27	-0.22	1.00
At1g03220	homologous to carrot EDGP and tomato XEGIP	✓	✓	nd	nd		
At1g03230	homologous to carrot EDGP and tomato XEGIP	✓	✓	nd	nd		
At5g19110	homologous to carrot EDGP and tomato XEGIP	✓	✓	8.45	8.89	0.44	1.00
At1g47710	homologous to serpin (serine protease inhibitor)	✓	✓	8.63	8.45	-0.18	1.00
At1g17860	inhibitor family I3 (Kunitz-P family) (subfamily I3A unassigned peptidase inhibitor homologues, MEROPS)	✓	✓	9.53	9.73	0.20	1.00
At1g73260	inhibitor family I3 (Kunitz-P family) (subfamily I3A unassigned peptidase inhibitor homologues, MEROPS)	✓	✓	12.54	13.41	0.86	5.77E-05
At1g47540	inhibitor family I18 (mustard trypsin inhibitor-2 family) (family I18 unassigned peptidase inhibitor homologues, MEROPS)	✓	✓	nd	nd		
At2g40880	inhibitor family I25 (phytostatin) (cystatin family, I25.014, MEROPS)	✓	✓	nd	nd		
At5g05110	inhibitor family I25 (phytostatin) (cystatin family, I25.014, MEROPS)	✓	✓	8.48	8.95	0.47	1.00

At4g16500	inhibitor family I25 (cystatin family) (subfamily I25B unassigned peptidase inhibitor homologues, MEROPS)	✓	✓	9.36	9.60	0.24	1.00
At4g25260	homologous to plant invertase/ pectin methylesterase inhibitor (PMEI)	✓	✓	9.37	9.86	0.49	1.00
At5g46940	homologous to plant invertase/pectin methylesterase inhibitor	✓		6.63	6.57	-0.06	1.00
At5g46960	homologous to plant invertase/pectin methylesterase inhibitor	✓		nd	nd		
**proteases**							
At1g09750	homologous to aspartic protease (pepsin family) (Peptidase family A1, subfamily A1B unassigned peptidases, MEROPS)	✓	✓	nd	nd		
At3g02740	homologous to aspartic protease (pepsin family) (Peptidase family A1, subfamily A1B unassigned peptidases, MEROPS)	✓		7.75	7.76	0.01	1.00
At3g52500	homologous to aspartic protease (pepsin family) (Peptidase family A1, subfamily A1B unassigned peptidases, MEROPS)	✓		9.46	8.75	-0.72	1.79E-02
At3g54400	homologous to aspartic protease (pepsin family) (Peptidase family A1, subfamily A1B unassigned peptidases, MEROPS)	✓	✓	10.30	9.85	-0.45	1.00
At5g07030	homologous to aspartic protease (pepsin family) (Peptidase family A1, subfamily A1B unassigned peptidases, MEROPS)	✓	✓	nd	nd		
At1g79720	homologous to aspartic protease (CND41 peptidase) (Peptidase family A1, subfamily A1.050 , MEROPS)	✓		8.96	8.54	-0.42	1.00
At5g10770	homologous to aspartic protease (CND41 peptidase) (Peptidase family A1, A01.050, MEROPS)	✓	✓	11.73	9.55	-2.17	0.00E+00
At1g47128	homologous to cysteine proteinase (papain family) (RD21A, Peptidase family C1, C01.064 MEROPS)	✓	✓	nd	nd		
At5g43060	homologous to cysteine proteinase (papain family) (RD21 peptidase, Peptidase family C1, C01.064 MEROPS)	✓	✓	nd	nd		
At4g01610	homologous to cysteine proteinase (papain family) (Cathepsin B, Peptidase family C1, C01.049, MEROPS)	✓	✓	12.70	12.50	-0.20	1.00
At4g36880	homologous to cysteine protease (papain family) (Peptidase family C1, Brassicain, C01.021 MEROPS)	✓		9.40	8.26	-1.14	6.74E-11
At3g02110	homologous to serine carboxypeptidase D (SCPL25) (Peptidase family S10, S10.005, MEROPS)	✓	✓	8.21	7.81	-0.41	1.00
At5g23210	homologous to serine carboxypeptidase (SCPL34) (Peptidase family S10, S10.005, MEROPS)	✓	✓	9.83	9.42	-0.41	1.00
At4g30610	homologous to serine carboxypeptidase (BRS1 - Brassinosteroid-Insensitive BRI suppressor 1) (Peptidase family S10, S10.015 MEROPS)	✓		7.54	7.36	-0.18	1.00
**structural proteins**							
At1g28290	proline-rich protein	✓	✓	11.98	11.89	-0.09	1.00
At5g14920	proline-rich protein	✓	✓	nd	nd		
At2g05580	glycine-rich protein	✓	✓	7.29	7.39	0.10	1.00
At4g13340	LRR-extensin (AtLRX3)	✓		6.61	6.66	0.05	1.00
At3g24480	LRR-extensin (AtLRX4)	✓	✓	10.26	10.33	0.08	1.00
At4g18670	LRR-extensin (AtLRX5)	✓	✓	7.38	7.17	-0.20	1.00

**signaling**							
At4g05200	homologous to receptor kinase (RLK, DUF26-1b subfamily)	✓		6.50	6.45	-0.05	1.00
**At5g55730**	fasciclin-like arabinogalactan protein (AtFLA1)	✓	✓	8.50	8.14	-0.36	1.00
**proteins related to lipid metabolism**							
At1g29670	homologous to lipase acylhydrolase (GDSL family)	✓	✓	nd	nd		
At1g54010	homologous to lipase/acylhydrolase (GDSL family)	✓		nd	nd		
At1g54030	homologous to lipase/acylhydrolase (GDSL family)	✓	✓	10.96	10.41	-0.54	1.00
At3g48460	homologous to lipase acylhydrolase (GDSL family)	✓	✓	9.03	8.11	-0.91	6.07E-06
At5g15720	homologous to lipase acylhydrolase (GDSL family)		✓	7.25	6.89	-0.35	1.00
At2g38530	non-specific lipid transfer protein 2 (LTP2)	✓	✓	nd	nd		
At5g23820	expressed protein (ML domain - MD-2-related lipid recognition domain)	✓		nd	nd		
At2g16001	expressed protein (lipid recognition domain)	✓		nd	nd		
**miscellaneous functions**							
At2g27190	homologous to purple acid phosphatase (PAP1)	✓	✓	11.34	11.09	-0.25	1.00
At3g07130	homologous to purple acid phosphatase	✓	✓	6.43	6.47	0.05	1.00
At5g34850	homologous to purple acid phosphatase	✓		12.30	11.94	-0.36	1.00
At4g29270	homologous to acid phosphatase	✓	✓	nd	nd		
At4g24340	homologous to phosphorylase	✓		nd	nd		
At3g02870	homologous to myo-inositol monophosphatase	✓		7.93	7.80	-0.13	1.00
At5g09440	homologous to *Nicotiana tabacum* phi-I	✓	✓	12.68	12.14	-0.54	1.00
At5g64260	homologous to *Nicotiana tabacum* phi-I	✓	✓	6.59	6.54	-0.05	1.00
At5g66590	homologous to *Nicotiana tabacum* pathogenesis-related protein PR1	✓	✓	6.48	6.41	-0.07	1.00
At2g28790	homologous to *Lycopersicon esculentum* osmotin	✓	✓	7.43	7.71	0.28	1.00
At5g15230	gibberellin-regulated protein (GASA4)	✓		13.16	13.93	0.77	2.75E-03
**At4g27110**	homologous to COBRA (AtCOBL10)	✓		6.95	6.73	-0.22	1.00
At1g09560	germin (subfamily 2, member 1, GLP5)		✓	11.34	11.06	-0.28	1.00
**unknown function**							
At3g56750	expressed protein		✓	nd	nd		
At3g22000	expressed protein (DUF26)		✓	nd	nd		
At1g26850	expressed protein (DUF248)		✓	nd	nd		
At1g80240	expressed protein (DUF642)	✓	✓	8.92	8.49	-0.43	1.00
At3g08030	expressed protein (DUF642)	✓	✓	12.40	12.06	-0.35	1.00
At4g32460	expressed protein (DUF642)	✓	✓	10.87	10.10	-0.77	2.20E-03
At5g11420	expressed protein (DUF642)	✓	✓	12.14	12.05	-0.09	1.00
At5g25460	expressed protein (DUF642)	✓	✓	9.86	9.42	-0.43	1.00
At1g78460	expressed protein (SOUL heme binding domain)		✓	6.57	6.54	-0.04	1.00
At2g04690	expressed protein (homologous to a human brain CREG protein)	✓	✓	9.61	9.55	-0.06	1.00
At2g15220	expressed protein (Plant Basic Secreted Protein domain)	✓	✓	nd	nd		
At2g34700	expressed protein (Ole el allergen domain)	✓	✓	11.53	9.27	-2.27	0.00E+00
At3g20370	expressed protein (MATH domain)	✓		11.42	10.92	-0.50	1.00
At2g28490	expressed protein (cupin domain)	✓	✓	9.21	9.43	0.22	1.00
At3g22640	expressed protein (cupin domain)	✓	✓	8.53	9.00	0.47	1.00
At4g36700	expressed protein (cupin domain)	✓		6.87	7.09	0.22	1.00
