# Supplementary material for: Cell wall biogenesis of Arabidopsis thaliana elongating cells: transcriptomics complements proteomics
Source: BMC Genomics. 2009 Oct 31;10:505. doi: 10.1186/1471-2164-10-505 (PMC2774874; doi:10.1186/1471-2164-10-505)

Additional file 6. RT-PCR analysis of the transcripts of some genes encoding CWPs identified through proteomics.

A. Quantitative analysis

Results of Q (quantitative)-PCR are expressed in number of Cts (cycle thresholds). *At1g49240* encoding an actin was used as a control. Results of transcriptomics are expressed as log_2 of signal intensity measured on CATMA arrays. Results of proteomics are indicated as follows: + means that the protein was identified; - means that the protein was not identified; nd means that the protein is not a cell wall protein. Note that the results of Q-PCR are consistent with CATMA results. The higher the level of transcripts measured on CATMA arrays, the lower the number of Cts measured by Q-PCR.

gene accession number	functional annotation	Q-PCR		transcriptomics (CATMA)		proteomics
		5-days Ct	11 days Ct	5-days log_2	11 days log_2	
**At1g49240**	actin 8	21.89	21.69	13.42	13.29	nd
**At5g11420**	expressed protein (DUF642)	23.68	23.61	12.14	12.05	+
**At1g28290**	AtAGP31	22.91	22.18	11.98	11.89	+
**At3g16850**	GH28 (polygalacturonase)	23.60	23.46	8.39	8.18	+
**At1g05850**	GH9	21.62	21.78	13.22	12.68	-
**At1g66180**	Asp protease	23.37	23.62	11.80	11.06	-
**At3g07130**	Purple acid phosphatase (AtPAP15)	27.00	27.07	6.43	6.47	+
**At1g10550**	GH16 (AtXTH33)	28.38	28.79	6.47	6.47	+

Semi-quantitative analysis

B. Genes with high or moderate level of transcripts in CATMA analysis and for which the proteins were identified by proteomics (+). *At1g49240* encoding an actin was used as a control. **C.** Genes with high level of transcripts in CATMA analysis but for which the proteins were not identified by proteomics (-); or with low level of transcripts in CATMA analysis for which the proteins were identified by proteomics (+). **D.** Genes with transcript level under background in CATMA analysis (lower than 6.83) and for which the proteins were identified by proteomics (+). PCR was run for 26 cycles for genes having high levels of transcripts in CATMA analysis (**B** and **C**), or for 30 cycles for genes having level of transcripts lower than background in CATMA analysis (**D**).

Sequences of primers are provided in Additional file 6. Note that PCR amplifications result in a single band at the expected size.

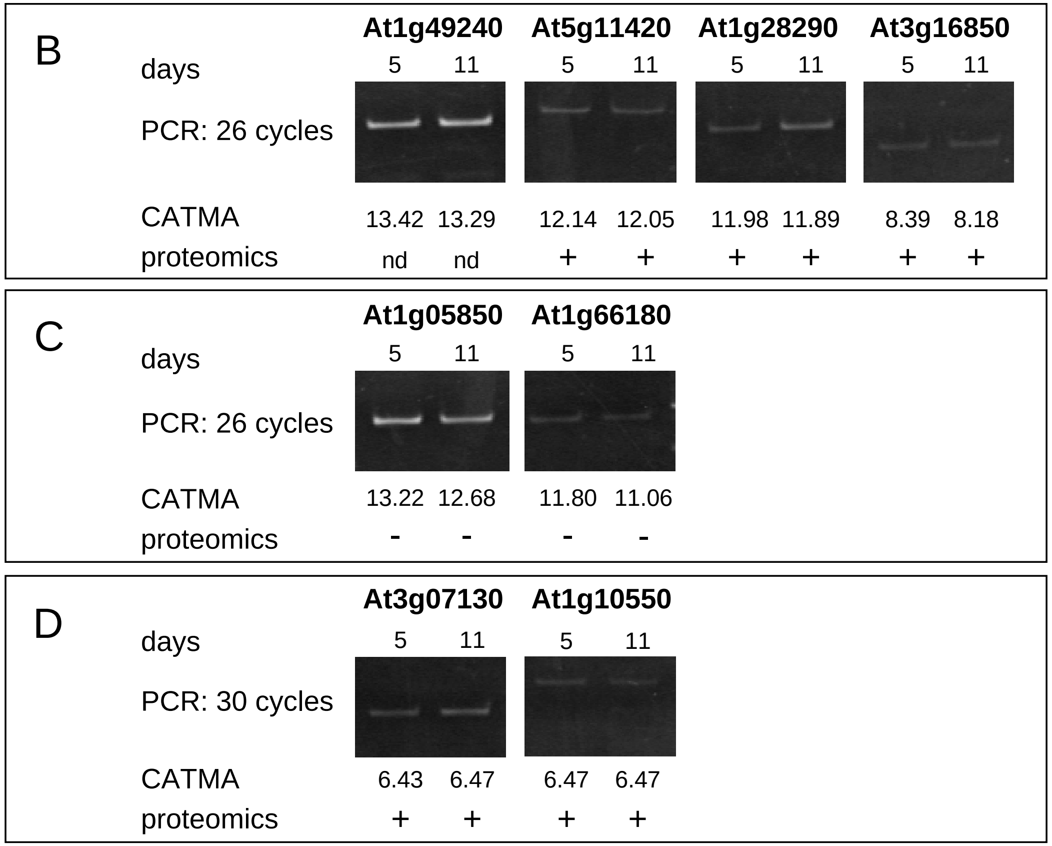

Supplement: Additional file 6 — RT-PCR analysis of the transcripts of some genes encoding CWPs identified through proteomics. The data provided represent the controls of microarray data by quantitative RT-PCR for some selected genes. [file 1471-2164-10-505-S6.pdf]
