# Supplementary material for: Cell wall biogenesis of Arabidopsis thaliana elongating cells: transcriptomics complements proteomics
Source: BMC Genomics. 2009 Oct 31;10:505. doi: 10.1186/1471-2164-10-505 (PMC2774874; doi:10.1186/1471-2164-10-505)
Supplement: Additional file 7 — Nucleotide primers used for PCR amplifications. The sequences of the oligonucleotide primers used for PCR analysis are listed in this file. [file 1471-2164-10-505-S7.pdf]

**Additional file 7. Nucleotide primers used for PCR amplifications**

| Accession number | Primer sequence (5'→3', sense orientation) | Primer sequence (5'→3', antisense orientation) | Length of expected fragment (bp) |
|---|---|---|---|
| At1g10550 | CAATTCAGTAAGATCGCCATTG | TTTGACACCAACCCAGCTC | 125 |
| At1g28290 | AACCATAAGACTCAAACCCTTC | TGAGGGTGGTGGTGATGAG | 95 |
| At1g49240 | CACCCGAGAGGAAGTACAGTG | CATACTCTGCCTTAGAGATCCACA | 93 |
| At1g66180 | ACCACAAGCTCAACAAATGGT | GGAGGAAGCTTTTTACGATGAC | 78 |
| At1g68560 | GTCGCCGTTAAATGTTGTTG | CCCATCTACATTGATGAAATCCT | 95 |
| At1g69530 | GCATCGCTCAATACAGAGC | GAGTGTCCGTTTATCGTAAACCTT | 97 |
| At2g21140 | TCTCATCCTTGATAAAGATGC | TCTACGTAGAATTCAACAAAGC | 111 |
| At2g28790 | GTGCCCACTACAACGGAAAA | TGAGCTAGAGAAGCTGGTGGT | 100 |
| At3g07130 | GGGATCGGCAACCTGATTA | GCCCATGTCTCGTTCTTCAT | 82 |
| At3g16850 | GTAGCCTTCAGAACCACCAGA | TCAACGCTTGTGAGATCGAC | 80 |
| At3g43270 | TTCCGATCTGCTACATTTGCT | GACCGCTTGGTGTTTTTCC | 96 |
| At4g12880 | GGCATGAAGCTAGATGTTTTAGTTG | ACATAAAATTGATTTCTTATTGTGCTG | 91 |
| At4g18670 | CACCACCTCCAATCTACGAAG | GCCCCTTTTGAGAACATTCTG | 112 |
| At5g05850 | TGGTACCACTGGAGGAAAGC | TTGCAACTCCATAGCCACAG | 95 |
| At5g11420 | GTCTCTTCTCTTTACTTTGGTCGTC | AGTCGCCGTTTGGTAACATC | 126 |
| At5g44360 | AGGAGCTTATTTGAATTACCGAGA | TCGCATCTTCAAAGCTCGTA | 74 |
| At5g64100 | GCAAGACTTCGCTGCTAAAAC | GCCGTTGAAGTTAACGAACC | 118 |
| desmin [a] | CAGCCTCAGTCCTCCAAATCACA | TAGGCCTGAGGTCACAGAGGT | |

a : Pig desmin RNA was used as an internal control for reverse transcription.
